# TRIM38 protects H9c2 cells from hypoxia/reoxygenation injury via the TRAF6/TAK1/NF-*κ*B signalling pathway

**DOI:** 10.7717/peerj.13815

**Published:** 2022-08-29

**Authors:** Zhengri Lu, Mengen Deng, Genshan Ma, Lijuan Chen

**Affiliations:** 1 Department of Cardiology, Zhongda Hospital, Southeast University, Nanjing, Jiangsu, China; 2 Department of Cardiology, Nanjing Lishui People’s Hospital, Zhongda Hospital Lishui Branch, Nanjing, Jiangsu, China

**Keywords:** TRIM38, Nuclear factor-κB, Hypoxia/reoxygenation, TAK1, Ubiquitination

## Abstract

Tripartite motif (TRIM) 38 is a ubiquitin E3 protein ligase that is involved in various intracellular physiological processes. However, the role of TRIM38 in myocardial ischaemia/reperfusion (I/R) injury remains to be elucidated. We aimed to establish an *in vitro* cellular hypoxia/reperfusion (H/R) model to explore the role and potential mechanisms of TRIM38 in H9c2, a rat cardiomyoblast cell line. Recombinant adenoviruses for silencing or overexpressing TRIM38 were constructed and transfected into H9c2 cells. Western blotanalysisshowed that TRIM38 expression was significantly decreased after H/R injury. Functionally, TRIM38 expression relieved inflammatory responses and oxidative stress, and inhibited H/R-induced apoptosis in H9c2 cells. Mechanistically, TRIM38 overexpression inhibited H/R-induced transforming growth factor beta-activated kinase 1 (TAK1)/nuclear factor-kappa B (NF-*κ*B) pathway activity in H9c2 cells. The opposite results were observed after TRIM38 knockdown. Furthermore, H/R-induced injury aggravated by TRIM38 deficiency in H9c2 cells was reversed upon treatment with 5Z-7-oxozeaenol, a TAK1 inhibitor. Therefore, TRIM38 reduction attenuated the anti-apoptotic capacity and anti-inflammatory potential of H/R-stimulated H9c2 cells by activating the TAK1/NF-*κ*B signalling pathway. Specifically, TRIM38 alleviated H/R-induced H9c2 cell injury by promoting TNF receptor-associated factor 6 degradation, which led to the inactivation of the TAK1/NF-*κ*B signalling pathway. Thus, our study provides new insights into the molecular mechanisms underlying H/R-induced myocardial injuries.

## Introduction

Myocardial infarction (MI) is a significant cause of disability and mortality globally (Thygesen et al., 2012). Reperfusion therapy after MI is primarily used to reduce MI size and improve its prognosis. However, restoring blood flow to the ischaemic myocardium may cause myocardial ischemia/reperfusion (I/R) injury (Yellon & Hausenloy, 2007). The pathogenesis of myocardial I/R injury is an intricate pathophysiological process involving numerous mechanisms, including oxidative stress, endoplasmic reticulum stress, inflammation, and autophagy (Kalogeris et al., 2012). Thus, in-depth studies to clarify the critical cellular and molecular mechanisms of I/R injury and develop new preventive prophylactic and therapeutic strategies are vital.

Ubiquitin ligase contributes to myocardial I/R injury by regulating various pathological processes (Wang et al., 2020; Tang et al., 2021). Our group previously reported that tripartite motif (TRIM) 8 and TRIM32, which are E3 ligase family members, participated in the regulation of pathological cardiac remodelling (Chen et al., 2016; Chen et al., 2017). TRIM38, an E3 ubiquitin ligase that contains three domains (RING, B-box/coiled coil, and SPRY), mediates inflammatory responses by targeting lysosome-dependent degradation of transforming growth factor (TGF)-beta-activated kinase 1 (TAK1) and MAP3K7-binding protein 2 (TAB2/3) and negatively regulates TLR3/4-mediated inflammatory responses *via* the proteasomal degradation system  (Hu et al., 2014; Hu et al., 2015). Furthermore, TRIM38 ameliorates IL-1*β*-mediated induction of chondrocyte apoptosis by regulating NF-*κ*B signals  (Hu et al., 2021). However, a possible mechanism by which TRIM38 protects cardiac cells against myocardial I/R injury has not yet been reported.

Nuclear factor-*κ*B (NF-*κ*B) is an essential transcription factor that regulates various biological processes, including immunity, inflammation, and apoptosis (Karin & Greten, 2005; Courtois & Gilmore, 2006). In unstimulated cells, NF-*κ*B is sequestered in the cytoplasm as a heterodimeric protein complexed with NF-*κ*B-alpha (I *κ*B *α*) inhibitors. When cells are stimulated, the NF-*κ*B kinase inhibitor I*κ*B kinase (IKK) is activated, resulting in the phosphorylation and degradation of I*κ*B*α*. Subsequently, the nuclear localisation signal on NF-*κ*B is exposed, leading to the nuclear translocation of NF-*κ*B and activation of transcription (Takaesu et al., 2003). Ubiquitination is a fundamental post-translational modification that plays an essential role in the NF-*κ*B pathway. Numerous TRIM members participate in the regulation of NF-*κ*B signals, indicating the importance of ubiquitination in maintaining fine regulation.

Several critical upstream signalling molecules are involved in IKK activation, including TNF receptor-associated factor 6 (TRAF6) and TAK1. TRAF6 is an E3 ubiquitin ligase containing a RING domain that recruits the TAK1-TAB complex by catalysing Lys63-linked polyubiquitin chain synthesis, which activates the endogenous TAK1 complex and downstream NF-*κ*B signalling pathways (Wang et al., 2001). TAK1 is an intracellular mitogen-activated protein kinase (MAP3K) necessary for activating the NF-*κ*B signalling pathway, as it is an upstream kinase of IKK (Hayden & Ghosh, 2008; Mihaly, Ninomiya-Tsuji & Morioka, 2014). TAK1/NF-*κ*B pathway regulates numerous physiological processes, including inflammation and apoptosis (Mihaly, Ninomiya-Tsuji & Morioka, 2014). Ubiquitin modifications play a crucial role in regulating TAK1-mediated NF-*κ*B activation. For instance, the ubiquitin E3 ligase, TRIM8, positively modulates TNF-*α*-and IL-1*β*-triggered NF-*κ*B activation by catalysing K63-linked polyubiquitination of TAK1 (Li et al., 2011). TRIM27 and TRIM29 negatively regulate NF-*κ*B activation by targeting TAB2 degradation (Dou et al., 2019; Chen et al., 2021). Additionally, TRIM38 negatively regulates NF-*κ*B and MAPK activation by controlling K48-linked ubiquitination of TRAF6 (Zhao et al., 2012). These findings suggest that TRIM proteins are essential regulators of TAK1/NF-*κ*B signals. Therefore, the functional identification of cellular TRIM38 might help in understanding the pathological mechanism of hypoxia/reperfusion (H/R) and exploring the relevance of TRIM38 as a therapeutic target in myocardial H/R injury.

Here, we identified TRIM38 as a novel repressor of TAK1 activation. TRIM38-mediated inactivation of NF-*κ*B may be associated with the TRAF6/TAK1 pathway. TRIM38 interacts with TRAF6 and specifically catalyses the polyubiquitination and degradation of TRAF6, resulting in suppressed TAK1 activation and downstream signal transduction. Based on these results, we elucidated a new molecular mechanism for H/R-mediated myocardial injury and identified novel therapeutic targets for MI.

## Material and Methods

### Cell culture

The H9c2 rat cardiomyoblast cell line, which is originally derived from embryonic rat heart tissues, was purchased from Zhong Qiao Xin Zhou Biotechnology Co., Ltd. (Shanghai, China). The cells were cultured in Dulbecco’s modified Eagle’s medium (Gibco, Shanghai, China) containing 10% foetal bovine serum (Gibco, Shanghai, China), at 37 °C and 5% CO_2_.

### *In vitro* establishment of H/R model

To simulate hypoxia/reoxygenation conditions, H9c2 cells were cultured in serum/glucose-free Dulbecco’s modified Eagle’s medium (Gibco, Shanghai, China) under hypoxia (94% N_2_, 5% CO_2_ and 1% O_2_) for 6 h and subsequently cultured in normal medium under reoxygenation (95% air and 5% CO_2_) for another 6 h. Control groups were cultured in normoxic conditions for 12 h.

### Adenovirus infection

Recombinant adenoviruses for silencing or overexpressing TRIM38 were purchased from GenePharma (Shanghai, China). Three shTRIM38 constructs were also obtained from GenePharma to generate three lines of AdshTRIM38 adenoviruses. The following sequences were used: shTRIM38-1: 5′-GCACAGTGAAGTCAAGAAAGG- 3′, shTRIM38-2: 5′-GGTGGAAGACTAGTGACTTAC-3′, and shTRIM38-3: 5′-GCTGTTGTTGGGAAGATCAGC-3′. H9c2 cells were infected with different adenoviruses in diluted medium at a multiplicity of infection of 100 for 24 h.

### Western blot analysis

Total protein was extracted from H9c2 cells using RIPA lysis buffer (Solarbio, Beijing, China) containing protease and phosphatase inhibitors. A BCA kit (Biosharp, Hefei, China) was used to measure the protein concentration. Total protein samples (50 µg) were separated by SDS–PAGE and transferred to methanol-preactivated PVDF membranes and then blocked overnight with 10% non-fat milk in tris-buffered saline at 4 °C for 2 h. The membranes were probed with primary antibodies including TRIM38 (dilution: 1:200; MA5-26235; Invitrogen), Bax (dilution: 1:5,000; 60267-1; Proteintech), Bcl-2 (dilution: 1:5,000; 26593-1-AP; Proteintech), caspase 3 (dilution: 1:1,000; #14220; CST), C-caspase3 (dilution: 1:1,000; #9661, CST), p-Ikk*α* (dilution: 1:1000; PA5-121282; Invitrogen), IKK*α* (dilution: 1:1,000; #2682; CST) p-p65 (dilution: 1:1,000; #3033; CST), p65 (dilution: 1:1,000; #8242; CST), IkB*α* (dilution: 1:500; AF5002; Affinity Biosciences), p-IkB*α* (dilution: 1:1,000, #2859; CST), TAK1 (dilution: 1:1,000; #5206; CST), p-TAK1 (dilution: 1:1,000; #9339; CST) overnight at 4 °C with gentle shaking. After washing three times with 1× tris-buffered saline tween (TBST) buffer, the membranes were incubated with HRP-conjugated goat anti-rabbit secondary antibodies (dilution: 1:5,000; SA00001-2; Proteintech) and HRP-conjugated goat anti-mouse secondary antibodies (dilution: 1:5,000; SA00001-1; Proteintech) for 2 h at room temperature.

### Lactate dehydrogenase (LDH) measurement

The LDH leak rate was detected using an LDH Cytotoxicity Assay Kit (Beyotime, Shanghai, China), following standard procedures according to the manufacturer’s instructions. The absorbance intensity at OD490 nm was measured using a microplate reader (Bio-Rad Laboratories Ltd., Shanghai, China).

### Malondialdehyde (MDA), superoxide dismutase (SOD), and glutathione peroxidase (GSH-Px) measurement

The levels of intracellular MDA, SOD, and GSH-Px were measured using corresponding assay kits and detected using a microplate reader.

### ELISA assay

Culture supernatants were collected and the concentrations of TNF-*α*, IL-1*β*, and IL-6 in cell supernatants were detected by enzyme-linked immunosorbent assay (ELISA) (Proteintech, Wuhan, China) according to the manufacturer’s instructions.

### Immunoprecipitation (IP) assay

The IP assay was performed as previously described (Chen et al., 2017). Briefly, cultured H9c2 cells were collected and lysed in IP buffer containing 20 mmol/L Tris–HCl (pH 8.0), 150 mmol/L NaCl, 0.5% NP-40, one mmol/L EDTA, and protease inhibitors. Cell lysates were incubated with protein A/G agarose beads for 3 h at 4 °C before blotting with primary antibodies.

### Ubiquitination assay

The ubiquitination assay was performed as previously described (Chen et al., 2017). Briefly, H9c2 cells were lysed in SDS lysis buffer containing 20 mmol/L Tris–HCl (pH 7.4), 150 mmol/L NaCl, one mmol/L EDTA, 1% SDS, and protease inhibitors. Then, the lysate was heated for 5 min to denature. The supernatant was diluted 10-fold with lysis buffer containing 20 mmol/L Tris–HCl (pH 7.4), 150 mmol/L NaCl, one mmol/L EDTA, 1% Triton X-100, and protease inhibitor cocktail. After centrifugation at 4 °C for 30 min, the supernatants were collected and subjected to IP with the indicated antibodies. The supernatants were then incubated with anti-ubiquitin antibodies to determine ubiquitination.

### Statistical analysis

Continuous variables are presented as the mean ± standard deviation (SD). The unpaired Student’s *t*-test was used for comparisons between two groups. One-way ANOVA followed by Bonferroni’s *post-hoc* test or Tamhane’s T2 post hoc test was used for comparisons among multiple groups. A two-tailed *P*-value of <0.05 was considered statistically significant. All statistical analyses were performed using GraphPad Prism Version 8.0 (GraphPad Prism Inc., San Diego, CA, USA) and IBM SPSS Version 25.0 (SPSS Inc., Chicago, IL, USA).

## Results

### H/R increased H9c2 apoptosis and reduced TRIM38 expression

**Figure 1:**
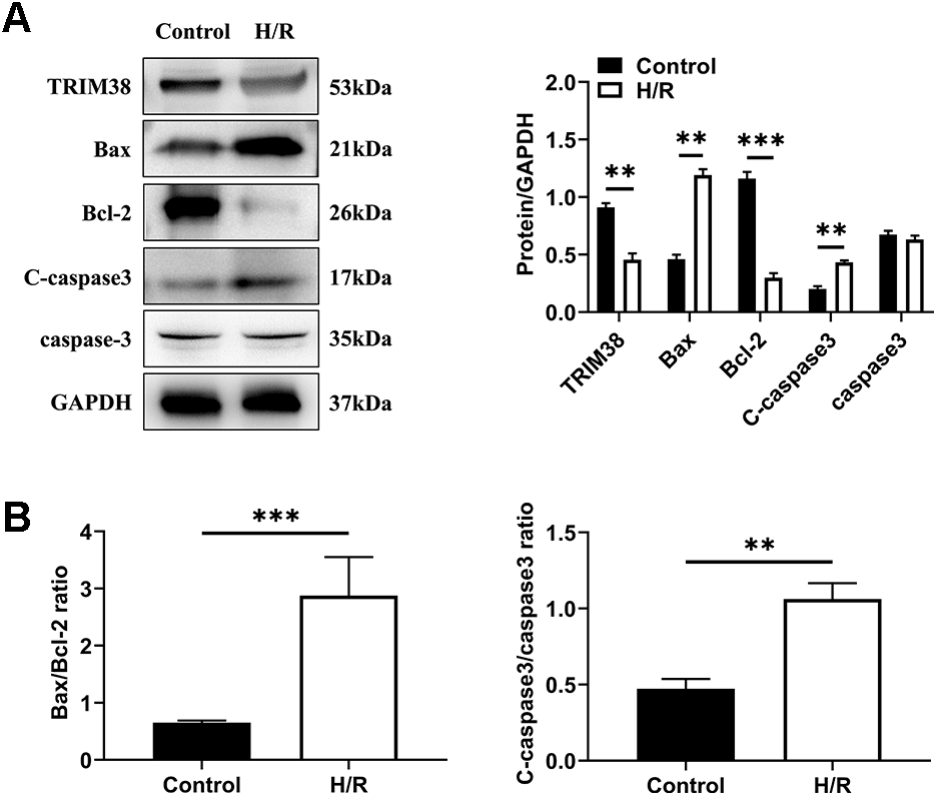
TRIM38 expression and its roles in H9c2 cells exposed to H/R. H9c2 cells were exposed to 6 h of hypoxia, followed by 6 h of reoxygenation. (A) Representative immunoblotting analysis and quantification of TRIM38, Bax, Bcl-2, C-caspase3, and caspase3 protein levels in H9c2 cells insulted by H/R or not. (B) Calculation of the ratio of Bax/Bcl2 and C-caspase3/caspase3. *n* = 3 for each group. **p < 0.01 and ***p < 0.001 *versus* control. Unpaired two-tailed student *t* test for A and B.

To investigate the role of TRIM38 expression in myocardial H/R injury, a cellular model of H/R injury was established. H9c2 cells were cultured under hypoxic conditions for 6 h and then under normoxic conditions for 6 h. We analysed the expression of apoptosis-related proteins in the control and H/R groups using western blotting. Compared with the control group, H/R increased the levels of pro-apoptotic proteins Bcl-2-associated X protein (Bax) and cleaved caspase-3 and decreased the levels of anti-apoptotic protein Bcl-2, leading to a higher bax/Bcl-2 ratio, which showed that H/R induced H9c2 cell apoptosis (Figs. 1A and 1B). Next, we examined the effects of H/R on TRIM38 expression in H9c2 cells. TRIM38 protein expression was remarkably downregulated by H/R compared to that in the control group (Fig. 1A). These results suggest that TRIM38 may be associated with H/R injury-induced H9c2 cell apoptosis.

### TRIM38 overexpression alleviated H/R-induced myocardial apoptosis

**Figure 2:**
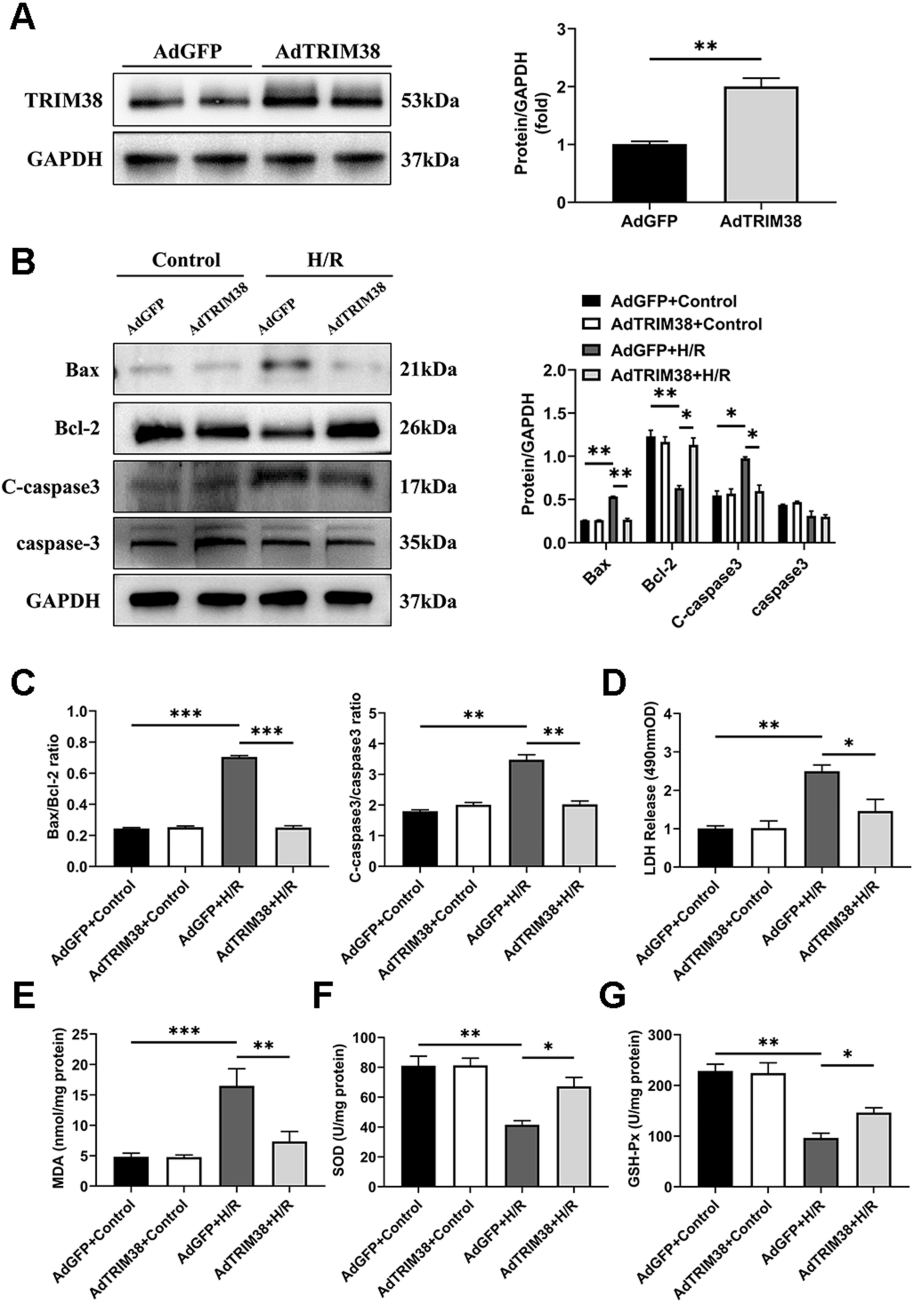
TRIM38 attenuates apoptosis and oxidative stress in H/R model. (A) Representative immunoblotting analysis (Left) and quantification (right) of TRIM38 expression in cultured H9c2 cells transfected with AdGFP or AdTRIM38. *n* = 4 for each group. (B) Representative immunoblotting analysis (Left) and quantification (right) of Bax, Bcl-2, C-caspase3, and caspase3 protein levels in H9c2 cells transfected with AdGFP or AdTRIM38 after H/R or not. (C) Calculation of the ratio of Bax/Bcl2 and C-caspase3/caspase3 in Control or H/R-treated H9c2 cells. (D) Effects of TRIM38 overexpression on LDH release in H/Rmodel. (E) Cellular MDA content in H/R model. (F) Cellular SOD activities in H/R model. (G) Cellular GSH-Px activities in H/R model. *n* = 3 for each group. *p < 0.05, **p < 0.01 and ***p < 0.001. Unpaired two-tailed student *t* test for A. One-way ANOVA followed by *post-hoc* tests for B–G.

To assess the effect of TRIM38 on H/R-induced apoptosis, we constructed a TRIM38 overexpressing adenovirus and transfected it into H9c2 cells, the AdGFP-infected H9c2 cells were used as controls (Fig. 2A). TRIM38 overexpression had little effect on the expression of the apoptosis markers under normoxic conditions. However, TRIM38 overexpression substantially decreased bax and cleaved caspase-3 expression, while increasing Bcl-2 expression, and thus reducing the bax/Bcl-2 ratio following H/R (Figs. 2B and 2C). The membrane integrity of damaged cells is disrupted, and the rate of LDH leakage can reflect the degree of cardiomyocyte damage. Subsequently, H/R markedly increased LDH release in H9c2 cells, whereas TRIM38 overexpression inhibited LDH release (Fig. 2D). Upon H/R, the excess production of ROS leads to oxidative stress and cellular damage. ROS lead to increased levels of MDA, which is the product of membrane lipid peroxidation. SOD and GSH-PX are the main enzymes for scavenging free radicals and preventing ROS accumulation. To analyse the impact of TRIM38 on cellular oxidative stress following H/R, we examined SOD and GSH-Px activity as well as MDA content. Overexpression of TRIM38 reduced the MDA content (Fig. 2E) and improved SOD and GSH-Px activity (Figs. 2F and 2G). These results demonstrate that TRIM38 protects against H/R injury.

### TRIM38 knockdown inhibits apoptosis in H9c2 cells after H/R

**Figure 3:**
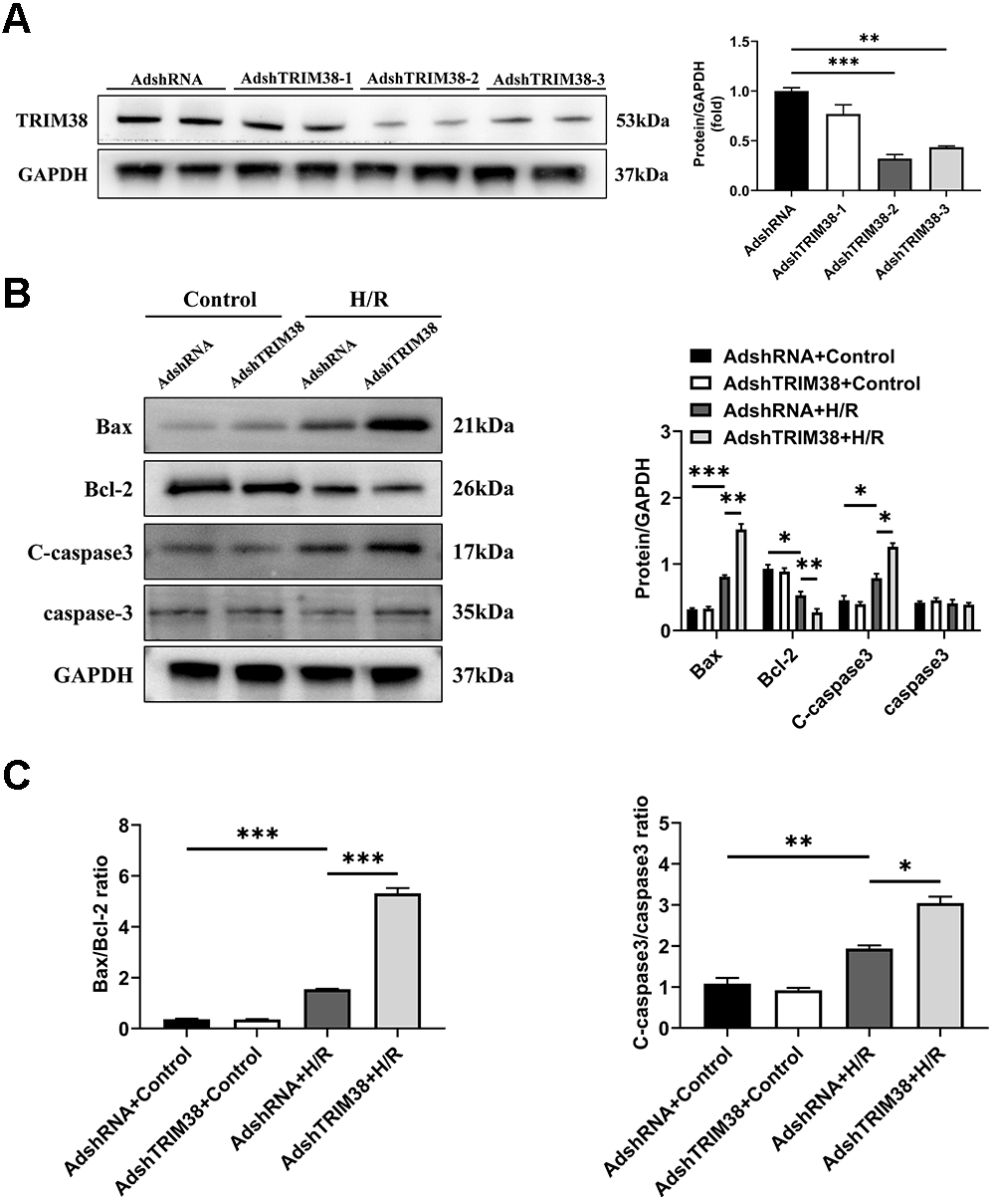
TRIM38 knockdown enhanced apoptosis in H9c2 Cells After H/R. (A) Representative immunoblotting analysis (Left) and quantification (right) of TRIM38 expression in cultured H9c2 cells transfected with AdshRNA or AdshTRIM38. (B) Representative immunoblotting analysis (Left) and quantification (right) of Bax, Bcl-2, C-caspase3, and caspase3 protein levels in H9c2 cells transfected with AdshRNA or AdshTRIM38 after H/R or not. (C) Calculation of the ratio of Bax/Bcl2 and C-caspase3/caspase3 in Control or H/R-treated H9c2 cells. *n* = 3 for each group. *p < 0.05, **p < 0.01 and ***p < 0.001. One-way ANOVA followed by *post-hoc* tests for A–C.

To confirm the effect of TRIM38 on H/R-induced H9c2 cell injury, we transfected H9c2 cells with a TRIM38 interference adenovirus, the AdshRNA-infected H9c2 cells were used as controls. As shown in Fig. 3A, compared with AdshRNA group, TRIM38 protein expression in H9c2 cells was downregulated by AdshTRIM38-1, AdshTRIM38-2, and AdshTRIM38-3. Compared with AdshTRIM38-1 and AdshTRIM38-3, AdshTRIM38-2 had a higher knockdown efficiency (Fig. 3A) and was therefore used in subsequent experiments. Conversely, TRIM38 knockdown promoted H/R-induced apoptosis by upregulating Bax and cleaved caspase-3 and downregulating Bcl2 expression; the Bax/Bcl2 ratio also increased (Figs. 3B and 3C).

### TRIM38 inhibits the NF-*κ*B signalling pathway to meditate inflammation during H/R injury

**Figure 4:**
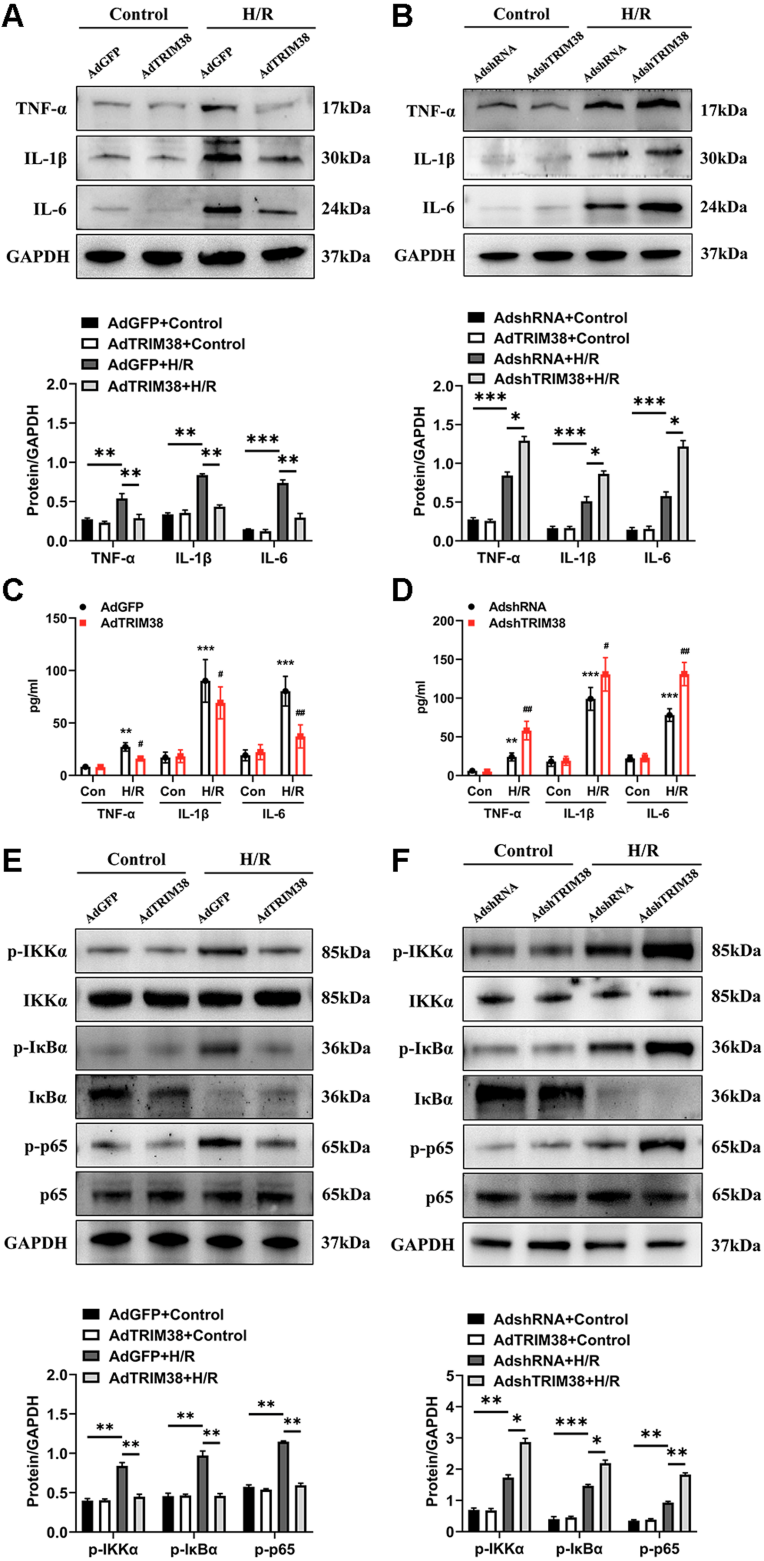
TRIM38 ameliorates inflammation in H/R-treated H9c2 cells *via* the NF-*κ* B pathways. (A) Representative immunoblotting analysis and quantification of proinflammatory cytokines in H9c2 cells transfected with AdGFP or AdTRIM38 after H/R stimuli or not. (B) Representative immunoblotting analysis and quantification of proinflammatory cytokines in H9c2 cells transfected with AdshRNA or AdshTRIM38 after H/R stimuli or not. (C–D) Evaluation of TNF-*α*, IL-1*β*, and IL-6 in the supernatants of cells using ELISA analysis . **p < 0.01 and ***p < 0.001 compared with Con/AdshRNA group and Con/AdGFP group, respectively; ^#^*p* < 0.05 and ^##^*p* < 0.01 compared with H/R/AdshRNA group and H/R/AdGFP group. (E–F) The protein expression of total and phosphorylated IKK*α*, I*κ*B*α*, and p65 in Control or H/R-treated H9c2 cells from the indicated groups. *n* = 3 for each group. *p < 0.05, **p < 0.01 and ***p < 0.001. One-way ANOVA followed by *post-hoc* tests for A–F.

An excessive inflammatory response is a major cause of H/R injury (Hu et al., 2016). The H/R induced the secretion of TNF-*α*, IL-1*β*, and IL-6, which was markedly downregulated by the overexpression of TRIM38 (Figs. 4A and 4C). In addition, the H/R-induced expression of TNF-*α*, IL-1*β*, and IL-6 was further promoted by TRIM38 knockdown (Figs. 4B and 4D). NF-*κ*B is a transcription factor and plays an important role in inflammation and apoptosis. To further explore the mechanisms of TRIM38 in myocardial H/R injury, we examined the activation of NF-*κ*B signalling in H9c2 cells after H/R. Western blot analysis showed that TRIM38 overexpression blocked the increase in p-IKK*α*, p-p65, and p-I*κ*B*α* levels that was induced by H/R (Fig. 4E). Furthermore, TRIM38 deficiency promoted NF-*κ*B signalling, as indicated by an increase in p-IKK*α*, p-p65, and p-I*κ*B*α* levels (Fig. 4F). These results show that TRIM38 inhibits the activation of NF-*κ*B signalling during H/R injury.

### TRIM38 triggers NF-*κ*B signalling by activating TAK1

**Figure 5:**
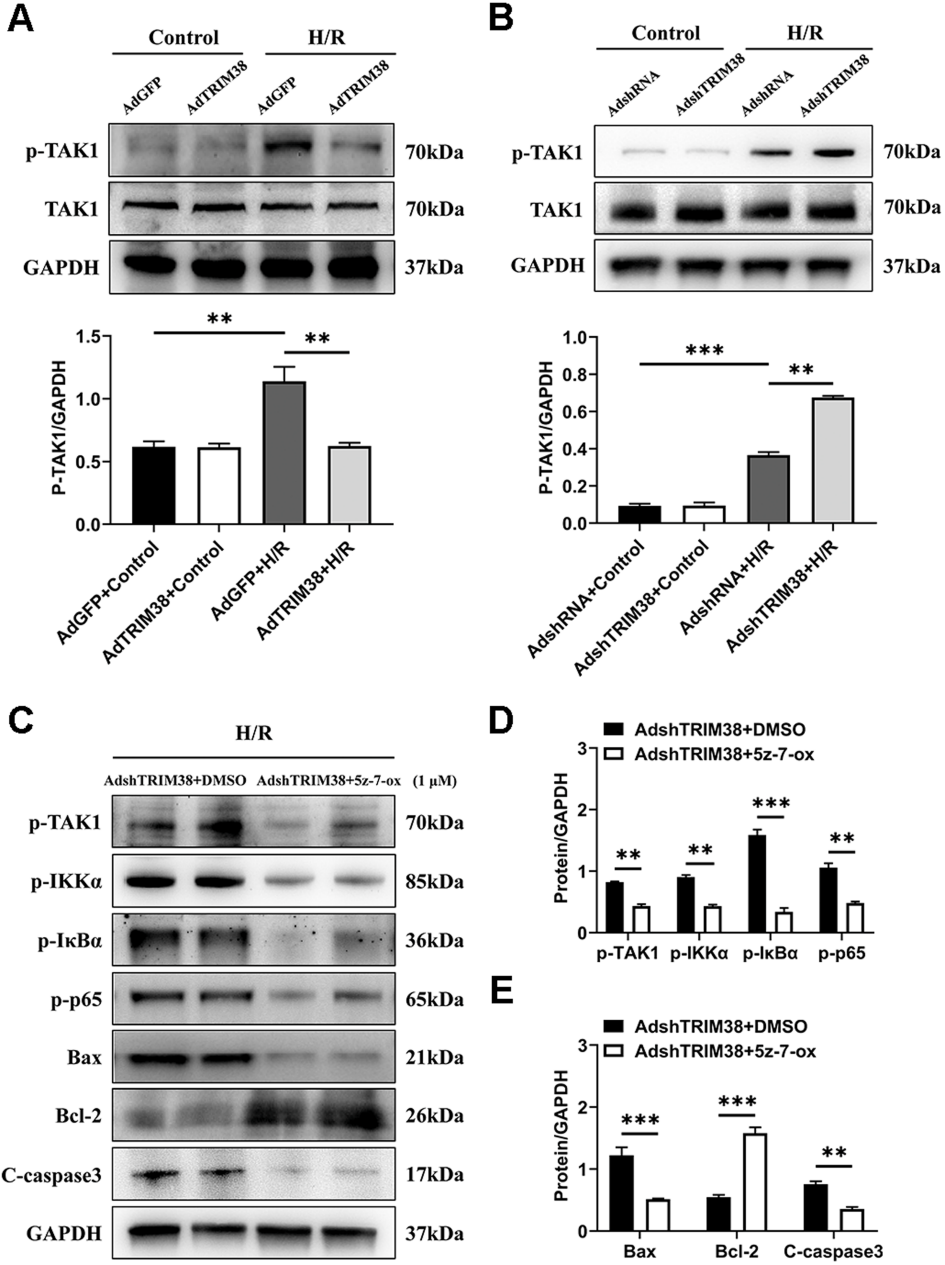
TRIM38 inhibited TAK1-NF-*κ*B signaling axis in H9c2 cells. (A–B) The protein expression of total and phosphorylated TAK1 in control or H/R-treated H9c2 cells from indicated groups. *n* = 3 for each group. (C–E) Representative immunoblottinganalysis and quantification of p-TAK1, p-IKK*α*, p-I*κ*B*α*, and p-p65 after pretreated with 5z-7-ox (1 µM) inH9c2 cells, and subjected to H/R for 12 h. *n* = 6 for each group. **p < 0.01 and ***p < 0.001. Unpaired two-tailed student *t* test for C–E. One-way ANOVA followed by *post-hoc* tests for A–B.

TAK1 is a critical upstream molecule for IKK activation and is activated by a broad range of stimuli (Mihaly, Ninomiya-Tsuji & Morioka, 2014; Mukhopadhyay & Lee, 2020). To assess whether TRIM38 inhibits TAK1 activation in H9c2 cells following H/R stimulation, TAK1 and phosphorylated TAK1 levels in H9c2 cells were determined using western blotting. The results showed that TRIM38 overexpression significantly attenuated H/R-enhanced phosphorylation of TAK1, whereas TRIM38 knockdown had the opposite effect (Figs. 5A and 5B). We used the TAK1 inhibitor 5Z-7-oxozeaenol to further explore its effect on the H/R-induced phosphorylation of TAK1. As shown in Figs. 5C and 5D, TRIM38 knockdown resulted in the H/R-induced upregulation of TAK1 and NF-*κ*B phosphorylation. The increase in TAK1 and NF-*κ*B phosphorylation following TRIM38 knockdown was markedly alleviated by 5Z-7-oxozeaenol (Figs. 5C and 5D). Furthermore, the inhibition of TAK1/NF-*κ*B signalling blocked the pro-apoptotic effects of TRIM38 knockdown in H/R-stimulated H9c2 cells. This was confirmed by the increased expression of Bcl2 and decreased expression of bax and caspase-3 proteins (Figs. 5C and 5E). Collectively, these results demonstrate that TRIM38 protects against myocardial I/R injury by suppressing NF-*κ*B activity *via* the regulation of the TAK1 signalling pathway.

### TRIM38 degraded TRAF6 *via* ubiquitination

**Figure 6:**
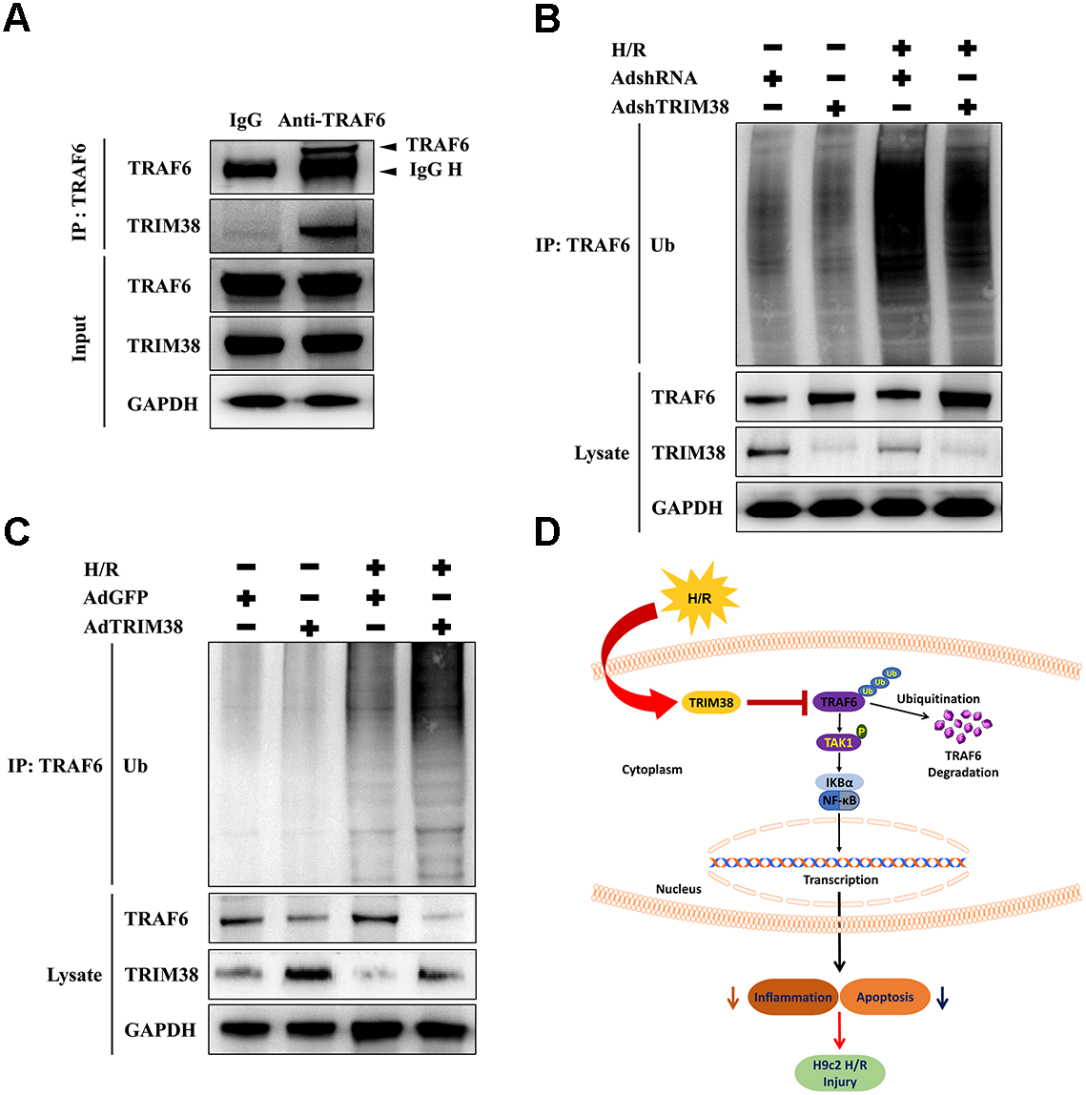
Under H/R conditions, TRIM38 degraded TRAF6 viaubiquitination. (A) H9c2 cells were subjected to H/R stimulation. Cell lysate and normal IgG antibody or TRAF6 antibody were subjected to Co-IP, and the indicated antibodies were used for immunoblotting analysis. (B–C) TRIM38 knockdown or overexpression cells were exposed with H/R. Immunoprecipitation assay was performed using lysates with anti-TRAF6 antibody. IB assay was performed using anti-TRAF6, anti-TRIM38, and anti-Ub antibodies. (D) Working model of TRIM38 protecting H9c2 cells from H/R injury by inhibiting TRAF6/TAK1/NF-*κ*B signaling pathway. TRIM38 alleviated H/R-induced H9c2 injury by promoting TRAF6 degradation, which led to the inactivation of TAK1, an upstream NF-*κ*B signalling molecule.

We explored the mechanism by which TRIM38 regulates the TAK1/NF-*κ*B pathway. TRAF6 interacts with downstream TAK1 and I*κ*B kinases to activate specific signalling pathways (Dong et al., 2006). As TRIM38 can modulate TRAF6 ubiquitination and degradation in response to LPS administration in macrophages (Zhao et al., 2012), we verified the interaction between endogenous TRIM38 and TRAF6 in H9c2 cells. As shown in Fig. 6A, TRAF6 co-precipitated with TRIM38 following H/R. Normal IgG, which was used as a control, did not co-precipitate with TRIM38. Next, we examined the effect of TRIM38 on the polyubiquitination and degradation of TRAF6. Western blot analysis showed that TRIM38 knockdown and overexpression markedly increased and decreased the baseline and post-H/R expression of TRAF6, respectively, compared with that in the control group (Figs. 6B and 6C). In response to H/R induction, ubiquitinated TRAF6 levels increased, but TRIM38 deficiency and overexpression decreased and enhanced polyubiquitinated TRAF6 levels, respectively (Figs. 6B and 6C).

## Discussion

The molecular mechanisms of myocardial I/R injury involve apoptosis, oxidative stress, imbalance in calcium homeostasis, and inflammation  (Sun et al., 2018). Unfolded or misfolded proteins resulting from oxidative stress, inflammatory responses, and cytotoxic events are ubiquitinated and transferred to the ubiquitin-proteasome system for degradation (Davies, 2001). Ubiquitination is an enzymatic post-translational modification, in which ubiquitin is attached to a substrate protein. Studies have demonstrated that ubiquitin E3 ligase is involved in inflammation and apoptosis during myocardial I/R injury (Patterson et al., 2007; Sun et al., 2019). In this study, we show, for the first time, that TRIM38 expression is significantly decreased in H9c2 cells following H/R injury. Functionally, TRIM38 relieved inflammatory responses and oxidative stress and inhibited apoptosis triggered by H/R. Mechanistically, TRIM38 protected H9c2 cells against H/R injury by regulating the TAK1/NF-*κ*B pathway. In particular, TRIM38 alleviated H/R-induced H9c2 cell injury by promoting TRAF6 degradation, which led to the inactivation of TAK1—an upstream NF-*κ*B signalling molecule (Fig. 6D).

Apoptosis is the principal factor associated with cardiomyocyte loss in I/R pathogenesis. Emerging evidence suggests that ubiquitination plays a pivotal role in myocardial I/R injury by regulating apoptosis (Li et al., 2020; Zhang et al., 2021). TRIM38 attenuates IL-1*β*-stimulated chondrocyte apoptosis  (Hu et al., 2021). In the present study, western blotting analysis suggested that TRIM38 expression was downregulated in cultured H9c2 cells subjected to H/R. Subsequently, the anti-apoptotic effect of TRIM38 on H9c2 cells during H/R was confirmed by functional experiments. Western blotting analysis indicated that TRIM38 overexpression notably protected H9c2 cells from H/R-induced apoptosis. In contrast, reduction of TRIM38 expression promotes apoptosis by regulating the Bax/Bcl-2 ratio and caspase-3 activity. These results preliminarily demonstrated that TRIM38 protected H9c2 cells from H/R-induced apoptosis.

Accumulating evidence suggests that oxidative stress and inflammatory response are critical factors associated with pathological process of myocardial I/R injury (Liu et al., 2021). Cellular reactive oxygen species (ROS) are major stressors of oxidative stress during myocardial ischaemia. Overproduction of oxygen free radicals exacerbates lipid peroxidation and inflammation, ultimately contributing to myocardial injury, particularly during reperfusion (Wu et al., 2020). Therefore, antioxidant and anti-inflammatory therapies may alleviate myocardial I/R injury to some degree (Hu et al., 2016; Liu et al., 2021). TRIM38 plays an important role in the regulation of inflammatory responses (Hu et al., 2014; Hu et al., 2015). Therefore, we explored whether TRIM38 overexpression could inhibit the production of inflammatory cytokines and oxidative stress substances. The levels of MDA and SOD reflect the degree of oxidative stress (Zhou et al., 2019). Our study revealed that TRIM38 overexpression significantly elevated the SOD levels, decreased the MDA levels, and inhibited the release of proinflammatory cytokines in H9c2 cells under conditions of H/R stimulation. These results indicate that TRIM38 attenuates H/R-induced H9c2 cell injury, partly by reducing the levels of inflammatory factors and inhibiting oxidative stress. In the pathological mechanism underlying myocardial I/R injury, inflammatory cells are the main cell type mediating inflammatory responses (Frangogiannis, 2012). Among them, monocytes and their progeny macrophages dominate cell infiltration and release inflammatory mediators and reactive oxygen species, thereby promoting inflammation and phagocytosis (Yellon & Hausenloy, 2007; Nahrendorf, Pittet & Swirski, 2010). Previous studies have demonstrated that TRIM38 inhibits TNF-*α*/IL-1 *β* signalling in mouse bone marrow-derived macrophages (Hu et al., 2015). However, it is unclear whether TRIM38 can inhibit NF-*κ*B-mediated pro-inflammatory responses in cardiomyocytes. Our findings suggest that TRIM38 can inhibit NF-*κ*B-mediated pro-inflammatory responses in cardiomyocytes. During myocardial I/R injury, cardiac-resident cells (*e.g.*, cardiomyocytes) can produce pro-inflammatory cytokines in response to various stimuli, thereby favouring a pro-inflammatory environment. However, given the dominant role of monocytes and macrophages in mediating inflammatory responses, exploring the link between TRIM38 and inflammatory cells and cardiomyocytes in myocardial tissues is worthy of further research (Fan et al., 2019; Algoet et al., 2022).

The NF-*κ*B family plays a critical role in numerous biological processes, including apoptosis and inflammatory responses (Karin & Greten, 2005; Courtois & Gilmore, 2006). When cardiomyocytes are stimulated by various chemical and mechanical signals, such as I/R, NF-*κ*B is activated (Xiong et al., 2021; Zhang et al., 2022). Therefore, we investigated the effect of TRIM38 on NF-*κ*B signalling. TRIM38 overexpression blocked the H/R-induced increase in the levels of p-IKK*α*, p-p65, and p-I*κ*B*α*. In contrast, TRIM38 knockdown displayed the opposite effects. These findings suggest that TRIM38 exerts protective effects on H/R-induced apoptosis and inflammation in H9c2 cells, at least in part, by inhibiting the NF-*κ*B signalling pathway. TAK1 linked cytokine stimulation with the activation of inflammatory signals. When stimulated by TGF-*β*, TNF-*α*, and IL-1, TAK1 phosphorylates and binds to the IKK complex, resulting in I*κ*B phosphorylation (Fan et al., 2010). This was further corroborated by the results of our *in vitro* rescue experiments. Treatment with a specific TAK1 inhibitor markedly attenuated the phosphorylation of TAK1 and the downstream signalling pathways in H9c2 cells subjected to H/R. Interestingly, we found that TRIM38 knockdown promoted H/R-induced apoptosis; however, the inhibitor 5Z-7-oxozeaenol blocked the effects caused by TRIM38 knockdown. In TRAF6-TAK1 pathway, TRAF6 ubiquitination promotes TAK1 activation. Therefore, we examined whether TRIM38 modulated the ubiquitination of TRAF6 in H9c2 cells. The results obtained from co-immunoprecipitation experiments showed that endogenous TRIM38 interacted with TRAF6, mediating its ubiquitination. Furthermore, we verified the negative correlation between TRIM38 and TRAF6 expression. An *in vivo* ubiquitination assay confirmed that TRIM38 deficiency and overexpression reduced and enhanced TRAF6 ubiquitination, respectively. Increased TRAF6 levels are involved in the activation of the TAK1/NF-*κ*B signalling pathway to regulate H9c2 cell injury.

Nevertheless, this study has a few limitations. First, we chose H/R-treated H9c2 cells rather than primary cardiomyocytes to explore the mechanism underlying the protective action of TRIM38. Although the H9c2 rat cardiomyoblast cell line demonstrates a reliable availability and consistency of responses, these cells may not fully reflect the interactions among primary cardiomyocytes. Moreover, the pathophysiological mechanisms underlying myocardial I/R injury are multifactorial and may involve factors other than inflammation and apoptosis. Furthermore, an *in vitro* model was constructed using standardised H/R conditions. Thus, additional experiments are needed to ascertain the protective effects of TRIM38 on cardiomyocytes under various hypoxic conditions.

In conclusion, we confirmed the novel cardioprotective effects of TRIM38 *in vitro*; these effects occur *via* the TRIM38-mediated blockage of the TRAF6/TAK1/NF-*κ*B signalling pathway, which suppresses apoptosis and inflammation in H/R-induced H9c2 cells. Furthermore, this study elucidated the molecular mechanism underlying the activity of TRIM38 in H/R-treated H9c2 cells and underscored a novel target for the alleviation of myocardial ischaemic injury.

##  Supplemental Information

10.7717/peerj.13815/supp-1 Supplemental Information 1 Protein bands

10.7717/peerj.13815/supp-2 Supplemental Information 2 Raw data of MOD protein

10.7717/peerj.13815/supp-3 Supplemental Information 3 Raw data of LDH Release (490 nm OD)

10.7717/peerj.13815/supp-4 Supplemental Information 4 Raw data of GSH-Px protein

10.7717/peerj.13815/supp-5 Supplemental Information 5 Evaluation of TNF-*α*, IL-1*β*, and IL-6 using ELISA

10.7717/peerj.13815/supp-6 Supplemental Information 6 Raw data of SOD protein

## Additional Information and Declarations

### Data Availability

The following information was supplied regarding data availability: The raw data is available in the Supplemental Files.
