# Supplementary figures and images for: TRIM38 protects H9c2 cells from hypoxia/reoxygenation injury via the TRAF6/TAK1/NF-κB signalling pathway (part 2 of 2)
Source: PeerJ. 2022 Aug 29;10:e13815. doi: 10.7717/peerj.13815 (PMC9435518; doi:10.7717/peerj.13815)

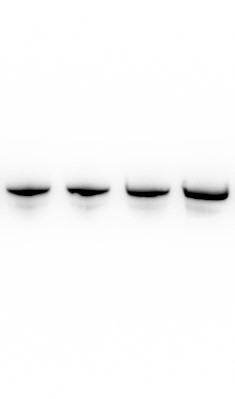

Supplement: Supplemental Information 1 [file peerj-10-13815-s001.zip › Raw data - Western blot bands/fig4/fig4E/p65/3.jpg]

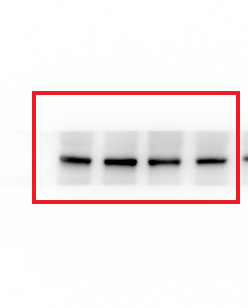

Supplement: Supplemental Information 1 [file peerj-10-13815-s001.zip › Raw data - Western blot bands/fig4/fig4F/GAPDH/GAPDH.jpg]

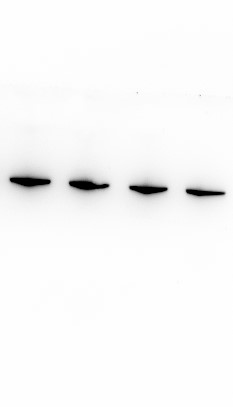

Supplement: Supplemental Information 1 [file peerj-10-13815-s001.zip › Raw data - Western blot bands/fig4/fig4F/GAPDH/GAPDH2.jpg]

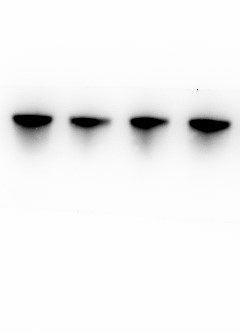

Supplement: Supplemental Information 1 [file peerj-10-13815-s001.zip › Raw data - Western blot bands/fig4/fig4F/GAPDH/GAPDH3.jpg]

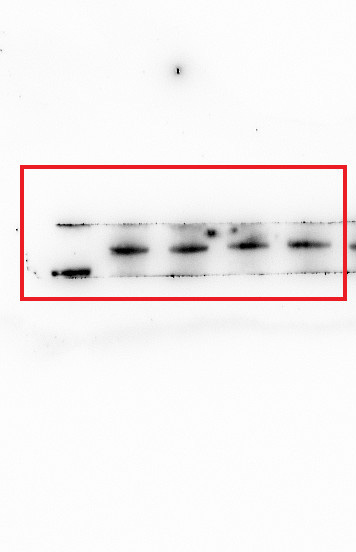

Supplement: Supplemental Information 1 [file peerj-10-13815-s001.zip › Raw data - Western blot bands/fig4/fig4F/IKK/1.jpg]

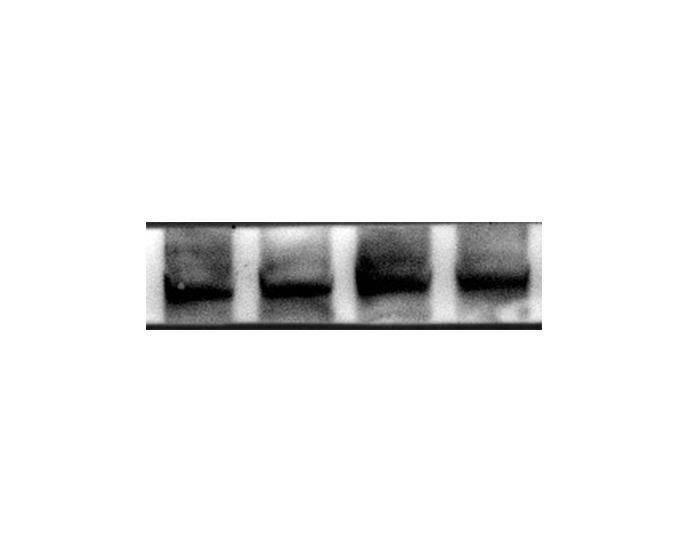

Supplement: Supplemental Information 1 [file peerj-10-13815-s001.zip › Raw data - Western blot bands/fig4/fig4F/IKK/2.tif]

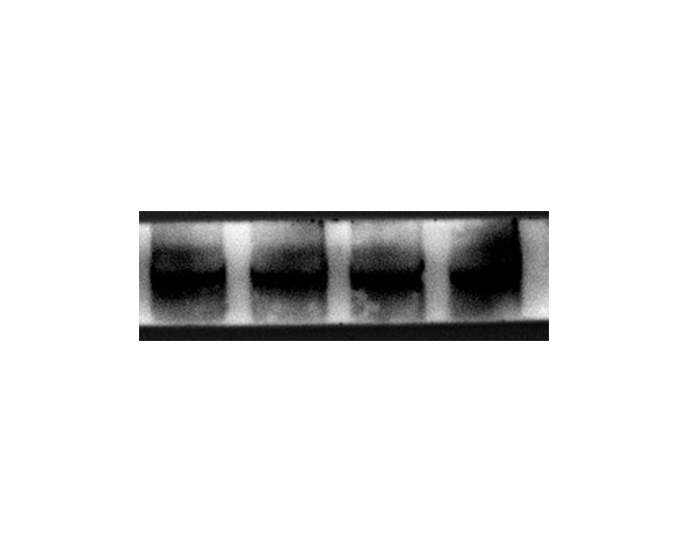

Supplement: Supplemental Information 1 [file peerj-10-13815-s001.zip › Raw data - Western blot bands/fig4/fig4F/IKK/3.tif]

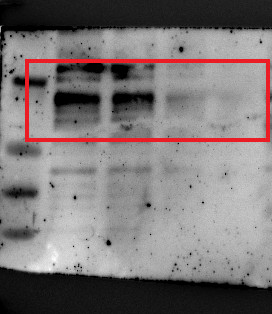

Supplement: Supplemental Information 1 [file peerj-10-13815-s001.zip › Raw data - Western blot bands/fig4/fig4F/IκBα/IκBα.jpg]

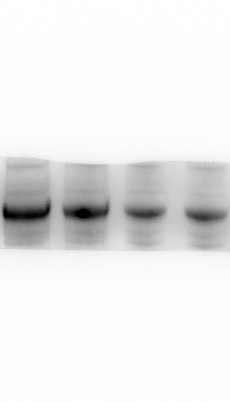

Supplement: Supplemental Information 1 [file peerj-10-13815-s001.zip › Raw data - Western blot bands/fig4/fig4F/IκBα/IκBα2.jpg]

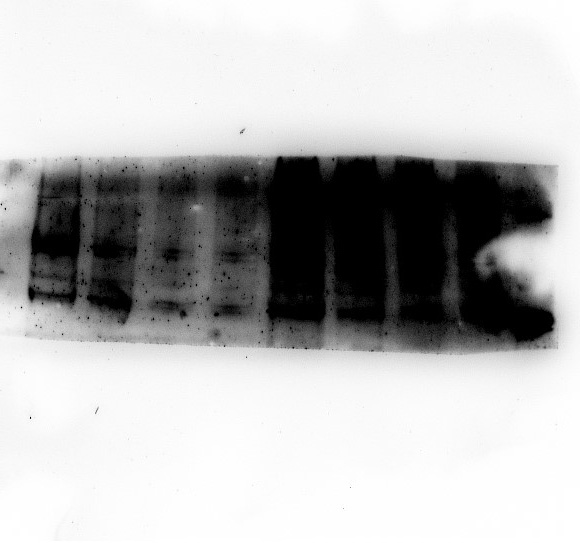

Supplement: Supplemental Information 1 [file peerj-10-13815-s001.zip › Raw data - Western blot bands/fig4/fig4F/IκBα/IκBα3.jpg]

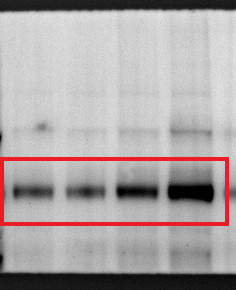

Supplement: Supplemental Information 1 [file peerj-10-13815-s001.zip › Raw data - Western blot bands/fig4/fig4F/p-IKK/1.jpg]

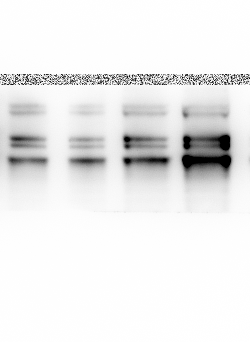

Supplement: Supplemental Information 1 [file peerj-10-13815-s001.zip › Raw data - Western blot bands/fig4/fig4F/p-IKK/2.tiff]

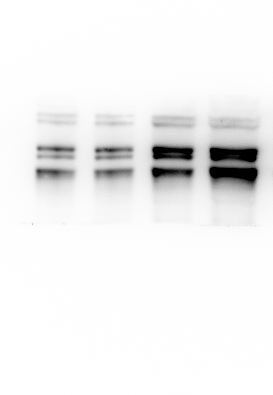

Supplement: Supplemental Information 1 [file peerj-10-13815-s001.zip › Raw data - Western blot bands/fig4/fig4F/p-IKK/3.tiff]

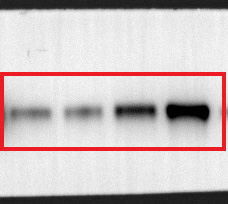

Supplement: Supplemental Information 1 [file peerj-10-13815-s001.zip › Raw data - Western blot bands/fig4/fig4F/p-IκBα/p-IκBα.jpg]

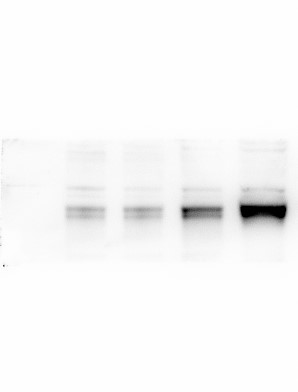

Supplement: Supplemental Information 1 [file peerj-10-13815-s001.zip › Raw data - Western blot bands/fig4/fig4F/p-IκBα/p-IκBα2.jpg]

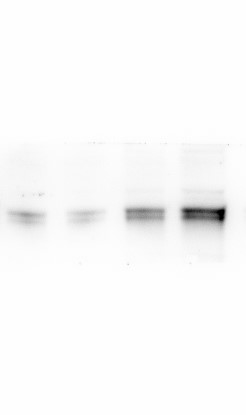

Supplement: Supplemental Information 1 [file peerj-10-13815-s001.zip › Raw data - Western blot bands/fig4/fig4F/p-IκBα/p-IκBα3.jpg]

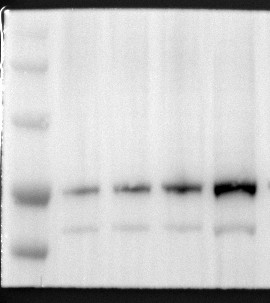

Supplement: Supplemental Information 1 [file peerj-10-13815-s001.zip › Raw data - Western blot bands/fig4/fig4F/p-p65/1.jpg]

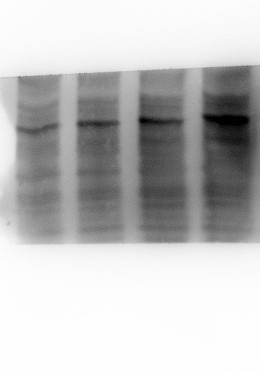

Supplement: Supplemental Information 1 [file peerj-10-13815-s001.zip › Raw data - Western blot bands/fig4/fig4F/p-p65/2.jpg]

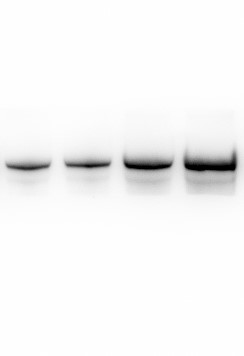

Supplement: Supplemental Information 1 [file peerj-10-13815-s001.zip › Raw data - Western blot bands/fig4/fig4F/p-p65/3.jpg]

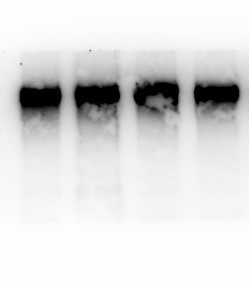

Supplement: Supplemental Information 1 [file peerj-10-13815-s001.zip › Raw data - Western blot bands/fig4/fig4F/p65/p65 2.jpg]

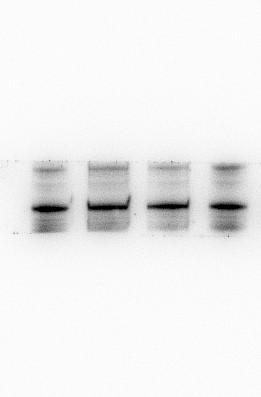

Supplement: Supplemental Information 1 [file peerj-10-13815-s001.zip › Raw data - Western blot bands/fig4/fig4F/p65/p65 3.jpg]

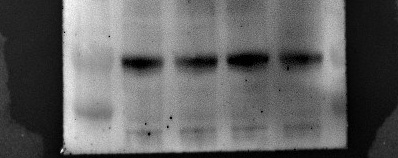

Supplement: Supplemental Information 1 [file peerj-10-13815-s001.zip › Raw data - Western blot bands/fig4/fig4F/p65/p65.jpg]

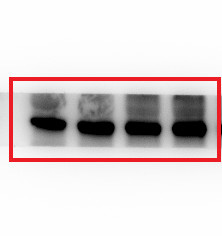

Supplement: Supplemental Information 1 [file peerj-10-13815-s001.zip › Raw data - Western blot bands/fig5/fig5A/GAPDH/GAPDH.jpg]

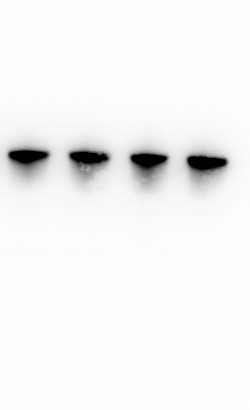

Supplement: Supplemental Information 1 [file peerj-10-13815-s001.zip › Raw data - Western blot bands/fig5/fig5A/GAPDH/GAPDH2.jpg]

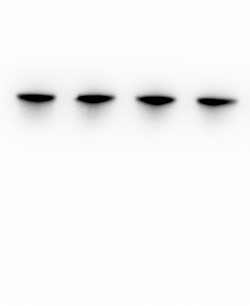

Supplement: Supplemental Information 1 [file peerj-10-13815-s001.zip › Raw data - Western blot bands/fig5/fig5A/GAPDH/GAPDH3.jpg]

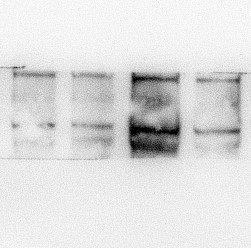

Supplement: Supplemental Information 1 [file peerj-10-13815-s001.zip › Raw data - Western blot bands/fig5/fig5A/p-TAK1/p-TAK1 2.jpg]

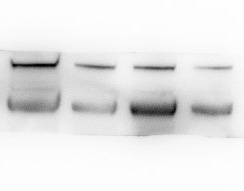

Supplement: Supplemental Information 1 [file peerj-10-13815-s001.zip › Raw data - Western blot bands/fig5/fig5A/p-TAK1/p-TAK1 3.jpg]

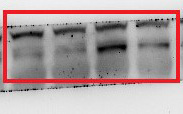

Supplement: Supplemental Information 1 [file peerj-10-13815-s001.zip › Raw data - Western blot bands/fig5/fig5A/p-TAK1/p-TAK1.jpg]

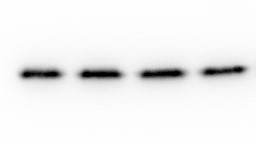

Supplement: Supplemental Information 1 [file peerj-10-13815-s001.zip › Raw data - Western blot bands/fig5/fig5A/TAK1/TAK1 2.jpg]

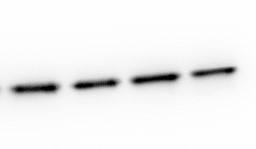

Supplement: Supplemental Information 1 [file peerj-10-13815-s001.zip › Raw data - Western blot bands/fig5/fig5A/TAK1/TAK1 3.jpg]

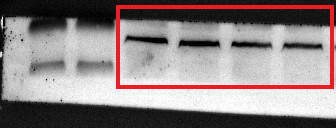

Supplement: Supplemental Information 1 [file peerj-10-13815-s001.zip › Raw data - Western blot bands/fig5/fig5A/TAK1/TAK1.jpg]

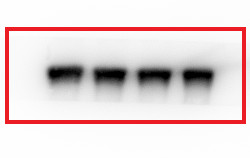

Supplement: Supplemental Information 1 [file peerj-10-13815-s001.zip › Raw data - Western blot bands/fig5/fig5B/GAPDH/GAPDH.jpg]

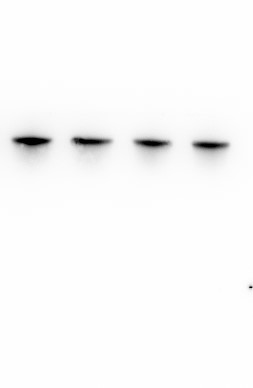

Supplement: Supplemental Information 1 [file peerj-10-13815-s001.zip › Raw data - Western blot bands/fig5/fig5B/GAPDH/GAPDH2.jpg]

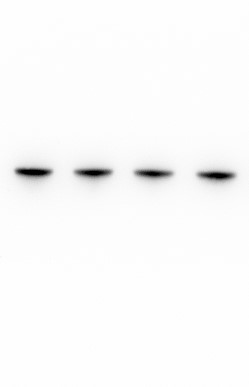

Supplement: Supplemental Information 1 [file peerj-10-13815-s001.zip › Raw data - Western blot bands/fig5/fig5B/GAPDH/GAPDH3.jpg]

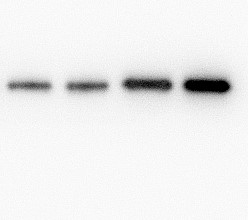

Supplement: Supplemental Information 1 [file peerj-10-13815-s001.zip › Raw data - Western blot bands/fig5/fig5B/p-TAK1/p-TAK1 2.jpg]

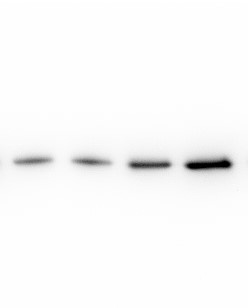

Supplement: Supplemental Information 1 [file peerj-10-13815-s001.zip › Raw data - Western blot bands/fig5/fig5B/p-TAK1/p-TAK1 3.jpg]

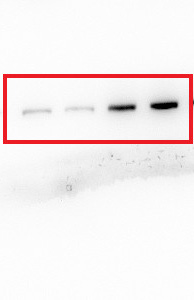

Supplement: Supplemental Information 1 [file peerj-10-13815-s001.zip › Raw data - Western blot bands/fig5/fig5B/p-TAK1/p-TAK1.jpg]

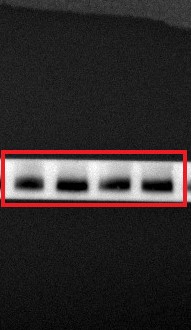

Supplement: Supplemental Information 1 [file peerj-10-13815-s001.zip › Raw data - Western blot bands/fig5/fig5B/TAK1/1.jpg]

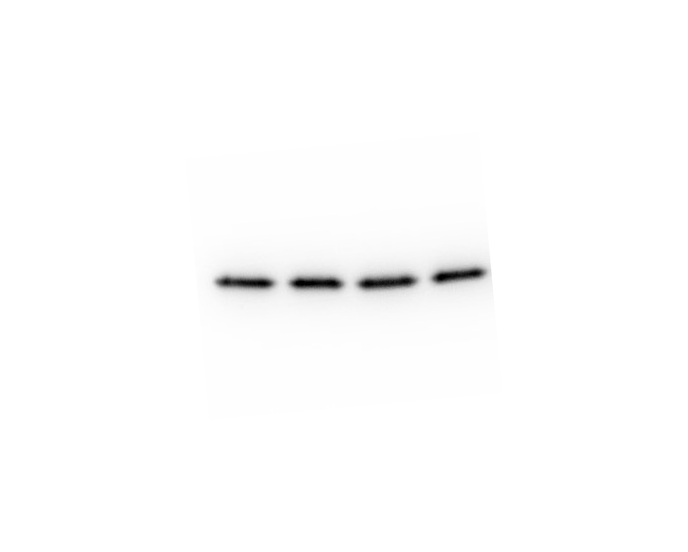

Supplement: Supplemental Information 1 [file peerj-10-13815-s001.zip › Raw data - Western blot bands/fig5/fig5B/TAK1/2.tif]

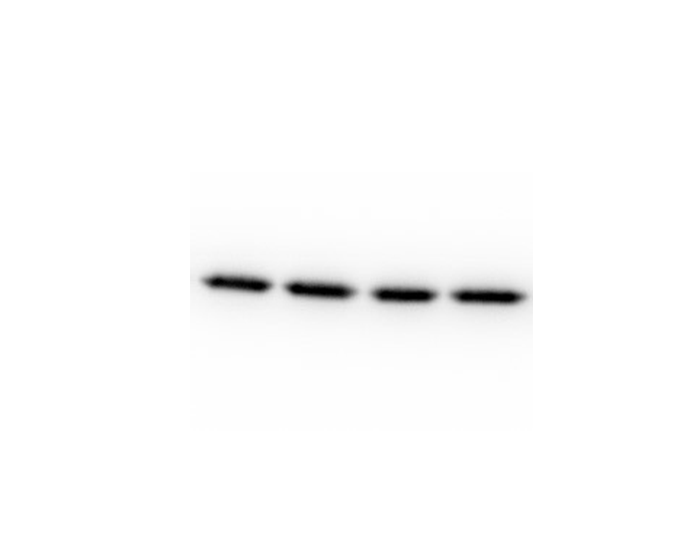

Supplement: Supplemental Information 1 [file peerj-10-13815-s001.zip › Raw data - Western blot bands/fig5/fig5B/TAK1/3.tif]

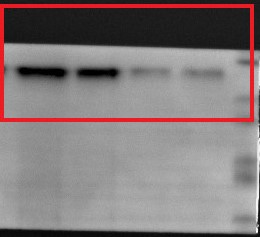

Supplement: Supplemental Information 1 [file peerj-10-13815-s001.zip › Raw data - Western blot bands/fig5/fig5C/bax/bax.jpg]

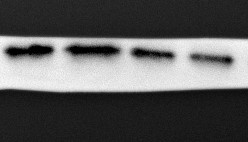

Supplement: Supplemental Information 1 [file peerj-10-13815-s001.zip › Raw data - Western blot bands/fig5/fig5C/bax/bax2.jpg]

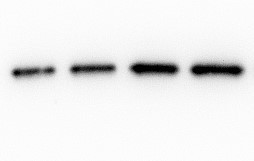

Supplement: Supplemental Information 1 [file peerj-10-13815-s001.zip › Raw data - Western blot bands/fig5/fig5C/bax/bax3.jpg]

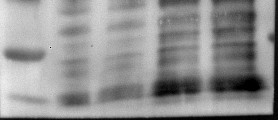

Supplement: Supplemental Information 1 [file peerj-10-13815-s001.zip › Raw data - Western blot bands/fig5/fig5C/bcl-2/1.jpg]

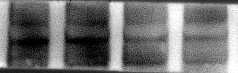

Supplement: Supplemental Information 1 [file peerj-10-13815-s001.zip › Raw data - Western blot bands/fig5/fig5C/bcl-2/2.jpg]

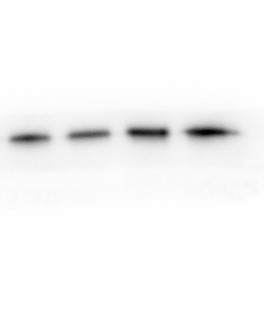

Supplement: Supplemental Information 1 [file peerj-10-13815-s001.zip › Raw data - Western blot bands/fig5/fig5C/bcl-2/3.jpg]

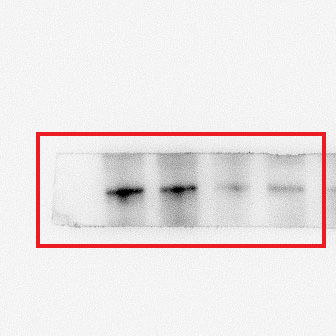

Supplement: Supplemental Information 1 [file peerj-10-13815-s001.zip › Raw data - Western blot bands/fig5/fig5C/c-caspase3/1.jpg]

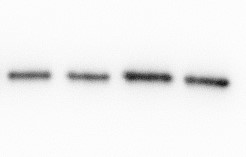

Supplement: Supplemental Information 1 [file peerj-10-13815-s001.zip › Raw data - Western blot bands/fig5/fig5C/c-caspase3/2.jpg]

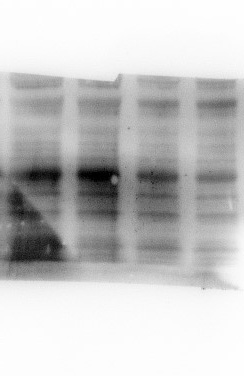

Supplement: Supplemental Information 1 [file peerj-10-13815-s001.zip › Raw data - Western blot bands/fig5/fig5C/c-caspase3/3.jpg]

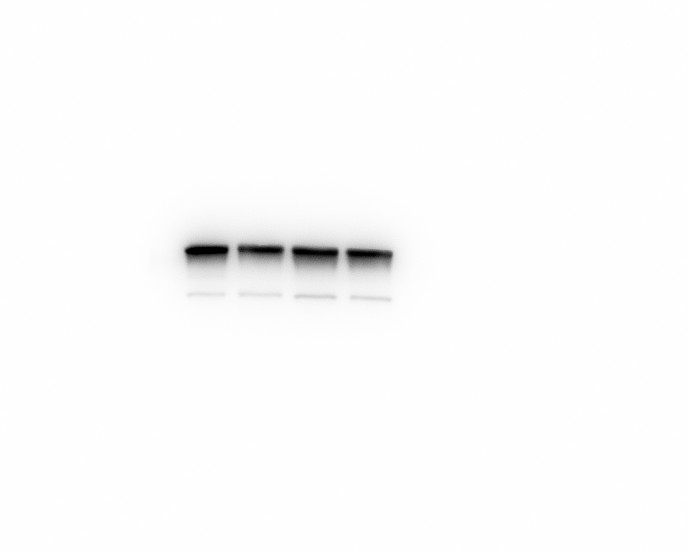

Supplement: Supplemental Information 1 [file peerj-10-13815-s001.zip › Raw data - Western blot bands/fig5/fig5C/GAPDH/GAPDH.jpg]

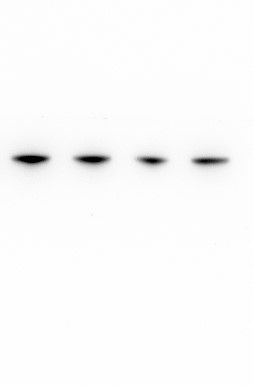

Supplement: Supplemental Information 1 [file peerj-10-13815-s001.zip › Raw data - Western blot bands/fig5/fig5C/GAPDH/GAPDH2.jpg]

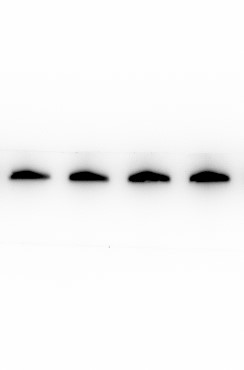

Supplement: Supplemental Information 1 [file peerj-10-13815-s001.zip › Raw data - Western blot bands/fig5/fig5C/GAPDH/GAPDH3.jpg]

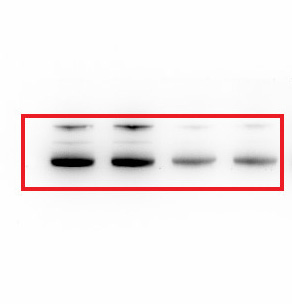

Supplement: Supplemental Information 1 [file peerj-10-13815-s001.zip › Raw data - Western blot bands/fig5/fig5C/p-IKKαβ/1.jpg]

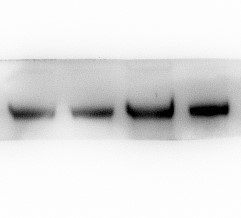

Supplement: Supplemental Information 1 [file peerj-10-13815-s001.zip › Raw data - Western blot bands/fig5/fig5C/p-IKKαβ/2.jpg]

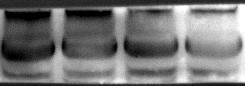

Supplement: Supplemental Information 1 [file peerj-10-13815-s001.zip › Raw data - Western blot bands/fig5/fig5C/p-IKKαβ/3.jpg]

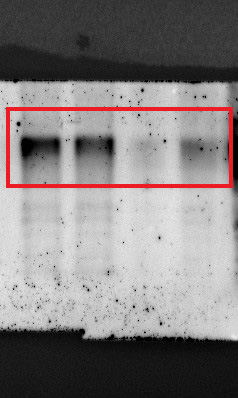

Supplement: Supplemental Information 1 [file peerj-10-13815-s001.zip › Raw data - Western blot bands/fig5/fig5C/p-IκBα/1.jpg]

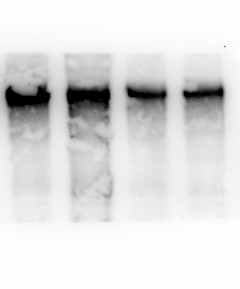

Supplement: Supplemental Information 1 [file peerj-10-13815-s001.zip › Raw data - Western blot bands/fig5/fig5C/p-IκBα/2.jpg]

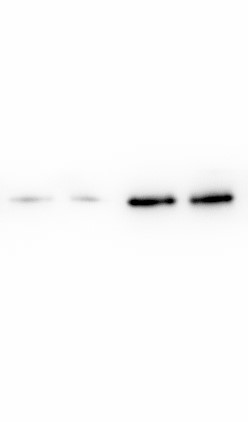

Supplement: Supplemental Information 1 [file peerj-10-13815-s001.zip › Raw data - Western blot bands/fig5/fig5C/p-IκBα/3.jpg]

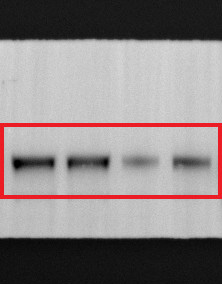

Supplement: Supplemental Information 1 [file peerj-10-13815-s001.zip › Raw data - Western blot bands/fig5/fig5C/p-p65/1.jpg]

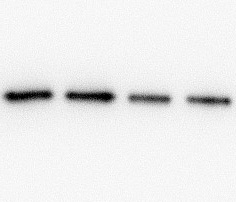

Supplement: Supplemental Information 1 [file peerj-10-13815-s001.zip › Raw data - Western blot bands/fig5/fig5C/p-p65/2.jpg]

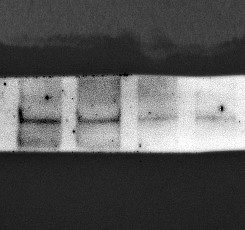

Supplement: Supplemental Information 1 [file peerj-10-13815-s001.zip › Raw data - Western blot bands/fig5/fig5C/p-p65/3.jpg]

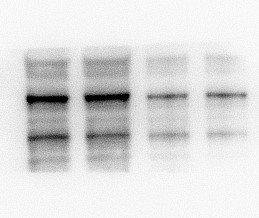

Supplement: Supplemental Information 1 [file peerj-10-13815-s001.zip › Raw data - Western blot bands/fig5/fig5C/p-TAK1/p-TAK1 2.jpg]

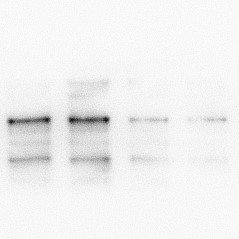

Supplement: Supplemental Information 1 [file peerj-10-13815-s001.zip › Raw data - Western blot bands/fig5/fig5C/p-TAK1/p-TAK1 3.jpg]

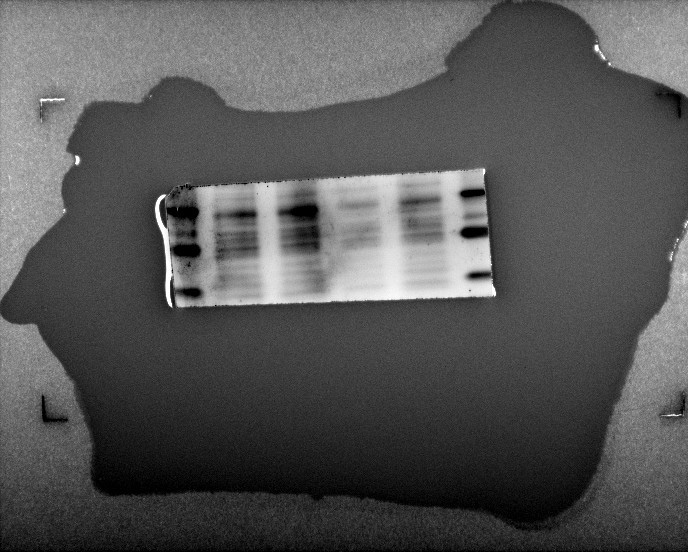

Supplement: Supplemental Information 1 [file peerj-10-13815-s001.zip › Raw data - Western blot bands/fig5/fig5C/p-TAK1/p-TAK1.jpg]

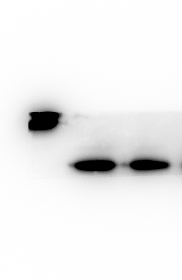

Supplement: Supplemental Information 1 [file peerj-10-13815-s001.zip › Raw data - Western blot bands/fig6/fig6A/GAPDH/1.tif]

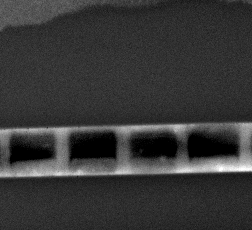

Supplement: Supplemental Information 1 [file peerj-10-13815-s001.zip › Raw data - Western blot bands/fig6/fig6A/GAPDH/2.tif]

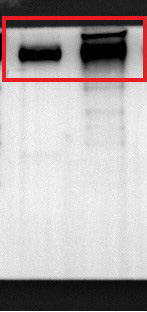

Supplement: Supplemental Information 1 [file peerj-10-13815-s001.zip › Raw data - Western blot bands/fig6/fig6A/IP TRAF6/1.jpg]

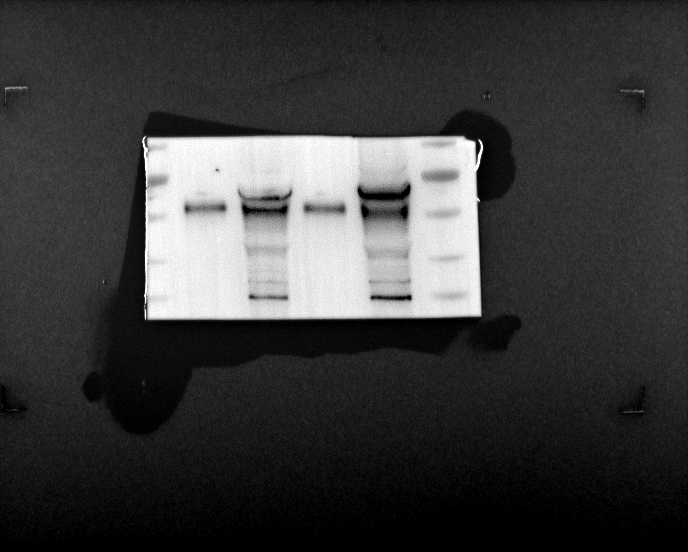

Supplement: Supplemental Information 1 [file peerj-10-13815-s001.zip › Raw data - Western blot bands/fig6/fig6A/IP TRAF6/2.Tif]

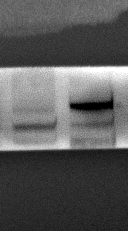

Supplement: Supplemental Information 1 [file peerj-10-13815-s001.zip › Raw data - Western blot bands/fig6/fig6A/IP TRIM38/1.tif]

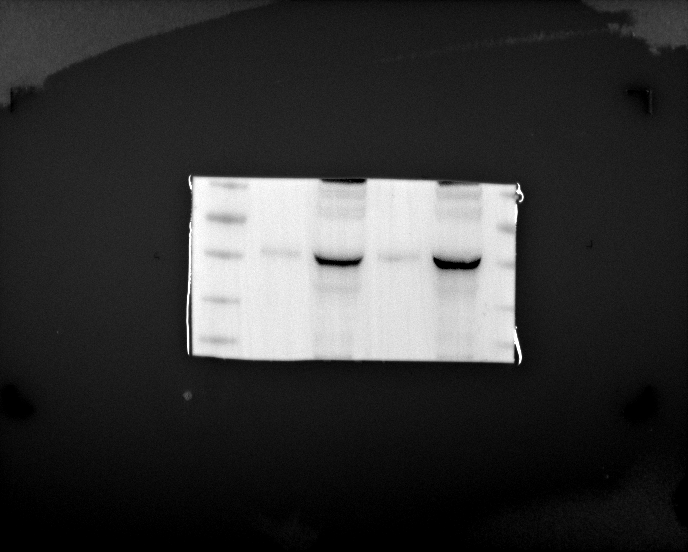

Supplement: Supplemental Information 1 [file peerj-10-13815-s001.zip › Raw data - Western blot bands/fig6/fig6A/IP TRIM38/2.Tif]

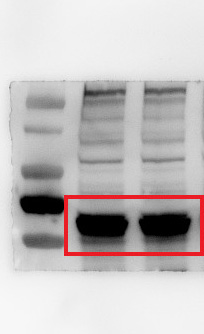

Supplement: Supplemental Information 1 [file peerj-10-13815-s001.zip › Raw data - Western blot bands/fig6/fig6A/TRAF6/1.jpg]

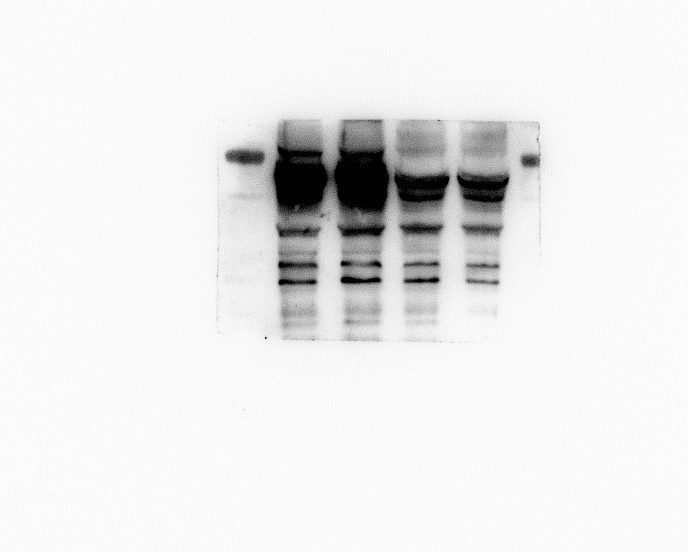

Supplement: Supplemental Information 1 [file peerj-10-13815-s001.zip › Raw data - Western blot bands/fig6/fig6A/TRAF6/2.Tif]

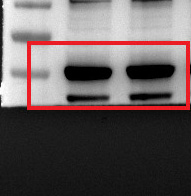

Supplement: Supplemental Information 1 [file peerj-10-13815-s001.zip › Raw data - Western blot bands/fig6/fig6A/TRIM38/1.jpg]

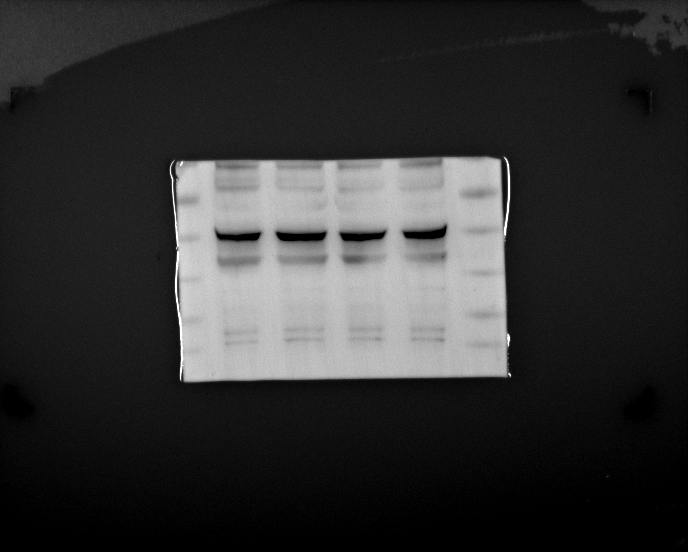

Supplement: Supplemental Information 1 [file peerj-10-13815-s001.zip › Raw data - Western blot bands/fig6/fig6A/TRIM38/2.Tif]

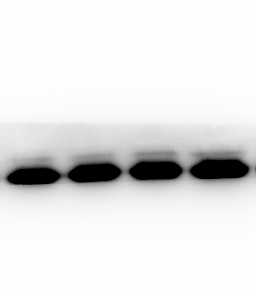

Supplement: Supplemental Information 1 [file peerj-10-13815-s001.zip › Raw data - Western blot bands/fig6/fig6B/GAPDH/1.tif]

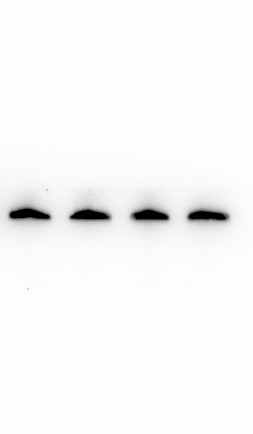

Supplement: Supplemental Information 1 [file peerj-10-13815-s001.zip › Raw data - Western blot bands/fig6/fig6B/GAPDH/2.jpg]

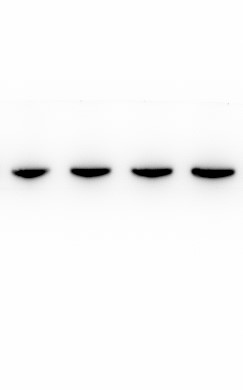

Supplement: Supplemental Information 1 [file peerj-10-13815-s001.zip › Raw data - Western blot bands/fig6/fig6B/GAPDH/3.jpg]

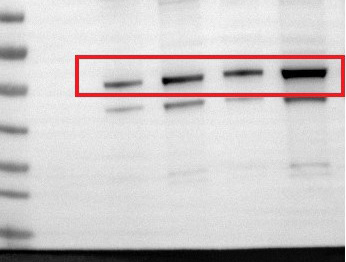

Supplement: Supplemental Information 1 [file peerj-10-13815-s001.zip › Raw data - Western blot bands/fig6/fig6B/TRAF6/1.jpg]

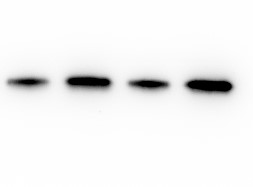

Supplement: Supplemental Information 1 [file peerj-10-13815-s001.zip › Raw data - Western blot bands/fig6/fig6B/TRAF6/2.jpg]

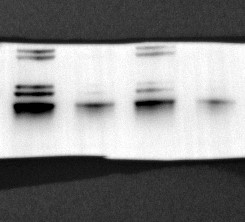

Supplement: Supplemental Information 1 [file peerj-10-13815-s001.zip › Raw data - Western blot bands/fig6/fig6B/TRAF6/3.jpg]

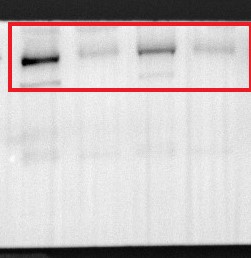

Supplement: Supplemental Information 1 [file peerj-10-13815-s001.zip › Raw data - Western blot bands/fig6/fig6B/TRIM38/1.jpg]

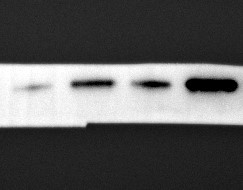

Supplement: Supplemental Information 1 [file peerj-10-13815-s001.zip › Raw data - Western blot bands/fig6/fig6B/TRIM38/2.jpg]

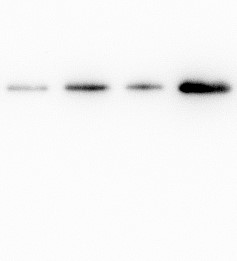

Supplement: Supplemental Information 1 [file peerj-10-13815-s001.zip › Raw data - Western blot bands/fig6/fig6B/TRIM38/3.jpg]

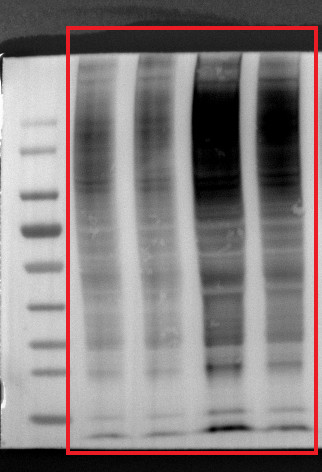

Supplement: Supplemental Information 1 [file peerj-10-13815-s001.zip › Raw data - Western blot bands/fig6/fig6B/Ub/1.jpg]

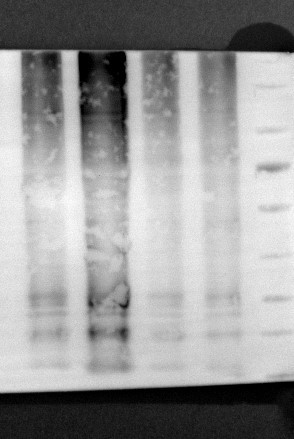

Supplement: Supplemental Information 1 [file peerj-10-13815-s001.zip › Raw data - Western blot bands/fig6/fig6B/Ub/2.jpg]

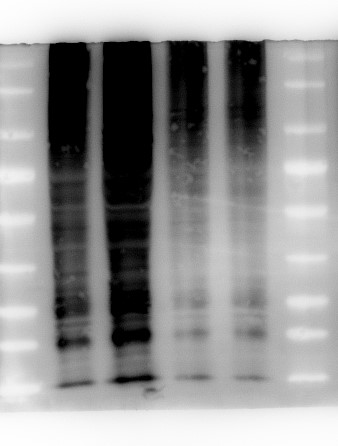

Supplement: Supplemental Information 1 [file peerj-10-13815-s001.zip › Raw data - Western blot bands/fig6/fig6B/Ub/3.jpg]

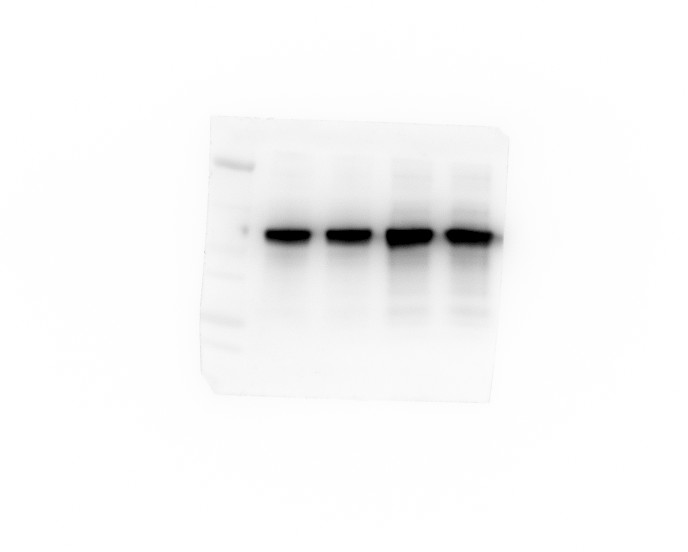

Supplement: Supplemental Information 1 [file peerj-10-13815-s001.zip › Raw data - Western blot bands/fig6/fig6C/GAPDH/1.jpg]

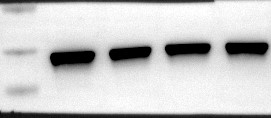

Supplement: Supplemental Information 1 [file peerj-10-13815-s001.zip › Raw data - Western blot bands/fig6/fig6C/GAPDH/2.jpg]

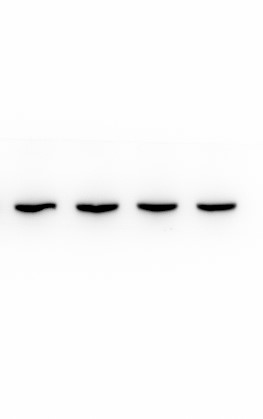

Supplement: Supplemental Information 1 [file peerj-10-13815-s001.zip › Raw data - Western blot bands/fig6/fig6C/GAPDH/3.jpg]

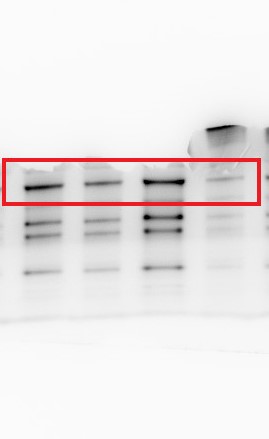

Supplement: Supplemental Information 1 [file peerj-10-13815-s001.zip › Raw data - Western blot bands/fig6/fig6C/TRAF6/1.jpg]

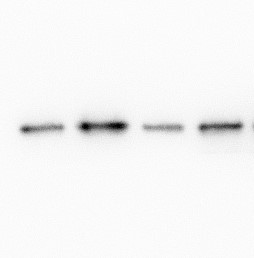

Supplement: Supplemental Information 1 [file peerj-10-13815-s001.zip › Raw data - Western blot bands/fig6/fig6C/TRAF6/2.jpg]

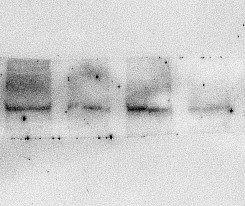

Supplement: Supplemental Information 1 [file peerj-10-13815-s001.zip › Raw data - Western blot bands/fig6/fig6C/TRAF6/3.jpg]

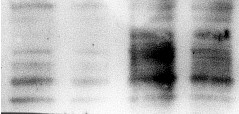

Supplement: Supplemental Information 1 [file peerj-10-13815-s001.zip › Raw data - Western blot bands/fig6/fig6C/TRIM38/1.jpg]

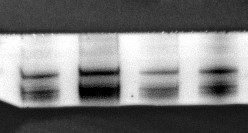

Supplement: Supplemental Information 1 [file peerj-10-13815-s001.zip › Raw data - Western blot bands/fig6/fig6C/TRIM38/2.jpg]

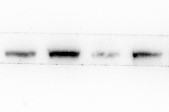

Supplement: Supplemental Information 1 [file peerj-10-13815-s001.zip › Raw data - Western blot bands/fig6/fig6C/TRIM38/3.jpg]

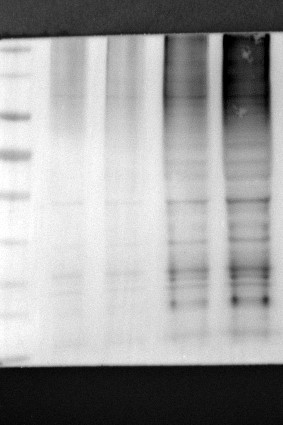

Supplement: Supplemental Information 1 [file peerj-10-13815-s001.zip › Raw data - Western blot bands/fig6/fig6C/Ub/1.jpg]

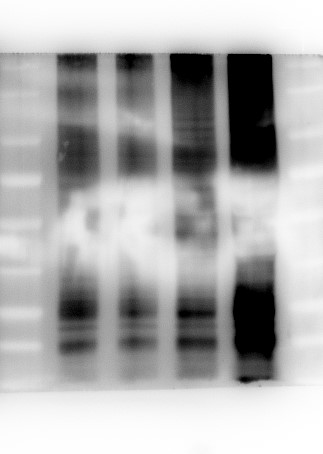

Supplement: Supplemental Information 1 [file peerj-10-13815-s001.zip › Raw data - Western blot bands/fig6/fig6C/Ub/2.jpg]

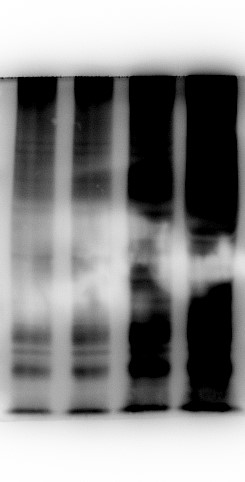

Supplement: Supplemental Information 1 [file peerj-10-13815-s001.zip › Raw data - Western blot bands/fig6/fig6C/Ub/3.jpg]
