# Supplementary figures and images for: TRIM38 protects H9c2 cells from hypoxia/reoxygenation injury via the TRAF6/TAK1/NF-κB signalling pathway (part 1 of 2)
Source: PeerJ. 2022 Aug 29;10:e13815. doi: 10.7717/peerj.13815 (PMC9435518; doi:10.7717/peerj.13815)

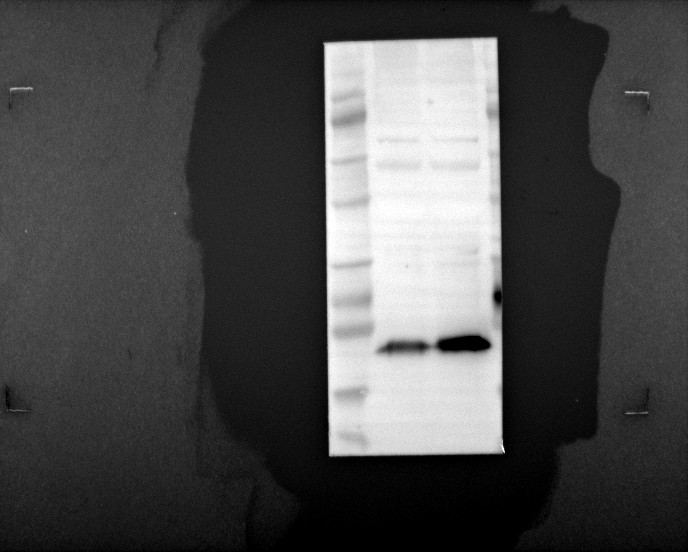

Supplement: Supplemental Information 1 [file peerj-10-13815-s001.zip › Raw data - Western blot bands/fig1/Bax/Bax1.jpg]

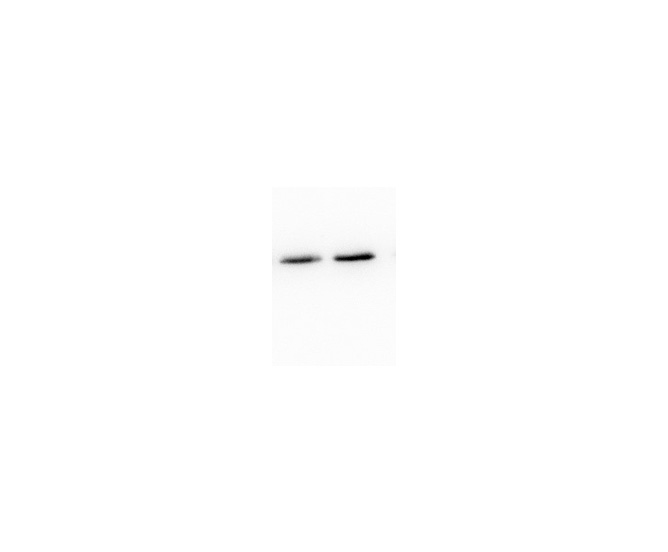

Supplement: Supplemental Information 1 [file peerj-10-13815-s001.zip › Raw data - Western blot bands/fig1/Bax/Bax2.jpg]

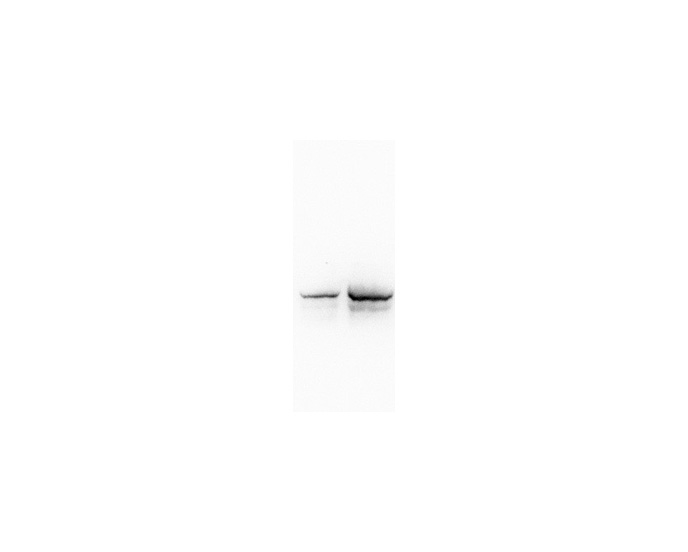

Supplement: Supplemental Information 1 [file peerj-10-13815-s001.zip › Raw data - Western blot bands/fig1/Bax/Bax3.jpg]

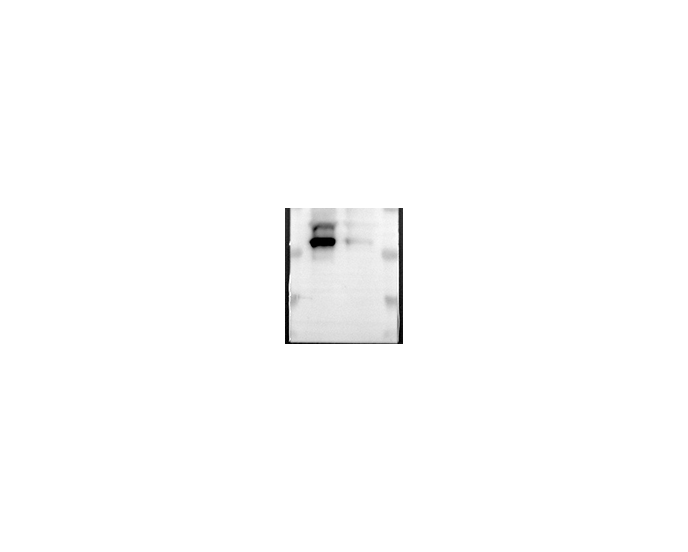

Supplement: Supplemental Information 1 [file peerj-10-13815-s001.zip › Raw data - Western blot bands/fig1/Bcl-2/Bcl-2 1.tif]

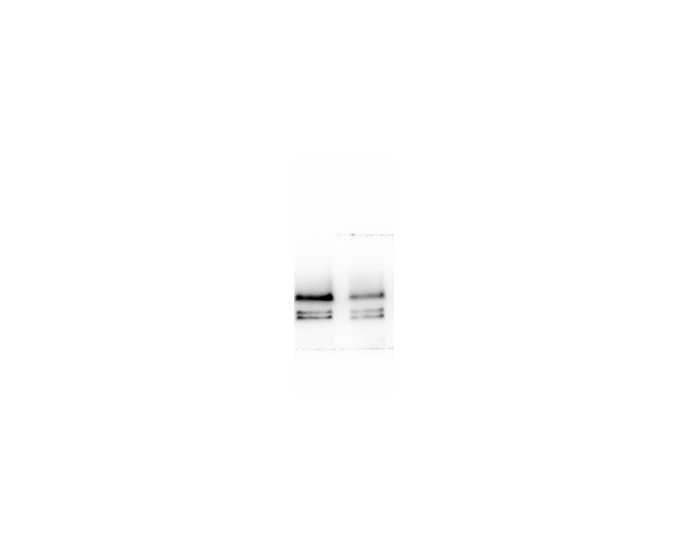

Supplement: Supplemental Information 1 [file peerj-10-13815-s001.zip › Raw data - Western blot bands/fig1/Bcl-2/Bcl-2 2.jpg]

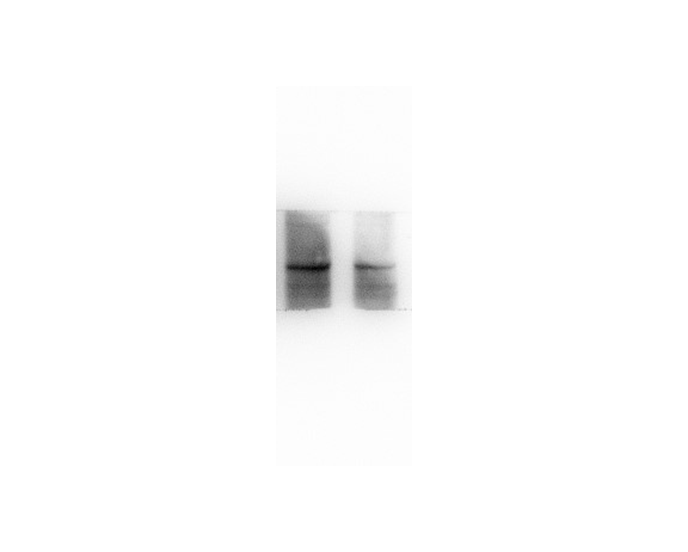

Supplement: Supplemental Information 1 [file peerj-10-13815-s001.zip › Raw data - Western blot bands/fig1/Bcl-2/Bcl-2 3.tif]

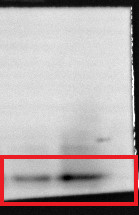

Supplement: Supplemental Information 1 [file peerj-10-13815-s001.zip › Raw data - Western blot bands/fig1/c-caspase3/c-caspase1.jpg]

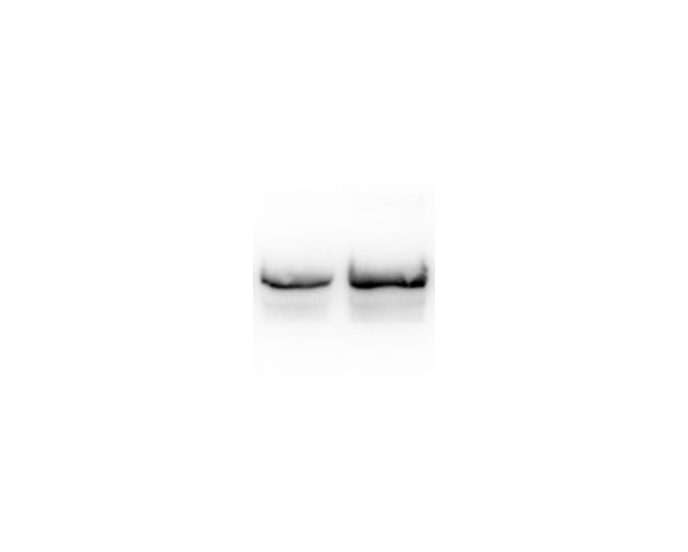

Supplement: Supplemental Information 1 [file peerj-10-13815-s001.zip › Raw data - Western blot bands/fig1/c-caspase3/c-caspase2.tif]

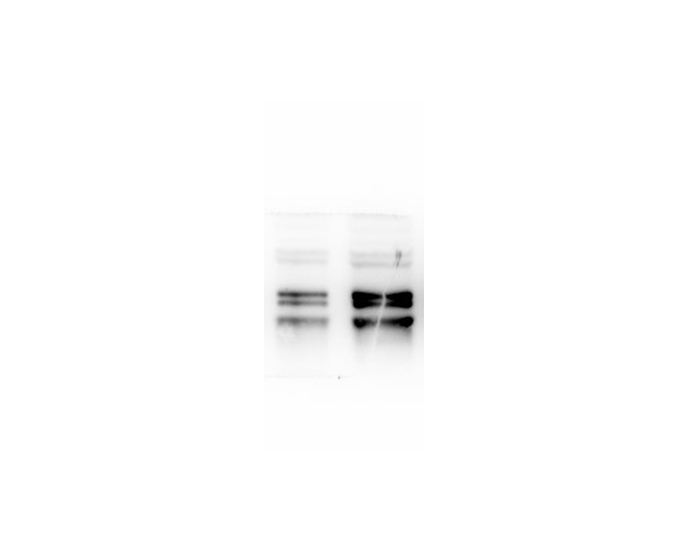

Supplement: Supplemental Information 1 [file peerj-10-13815-s001.zip › Raw data - Western blot bands/fig1/c-caspase3/c-caspase3.tif]

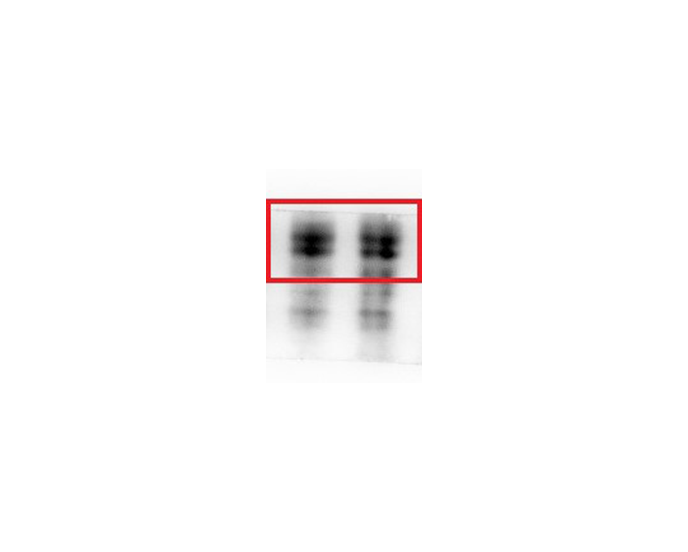

Supplement: Supplemental Information 1 [file peerj-10-13815-s001.zip › Raw data - Western blot bands/fig1/caspase3/caspase 1.tif]

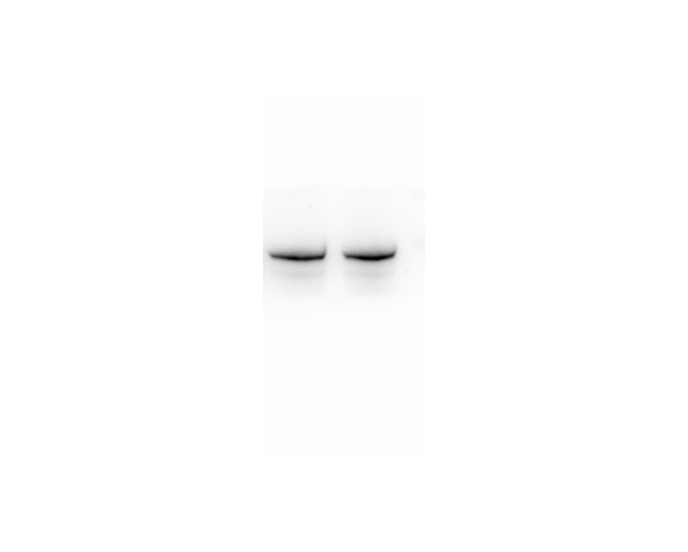

Supplement: Supplemental Information 1 [file peerj-10-13815-s001.zip › Raw data - Western blot bands/fig1/caspase3/caspase 2.tif]

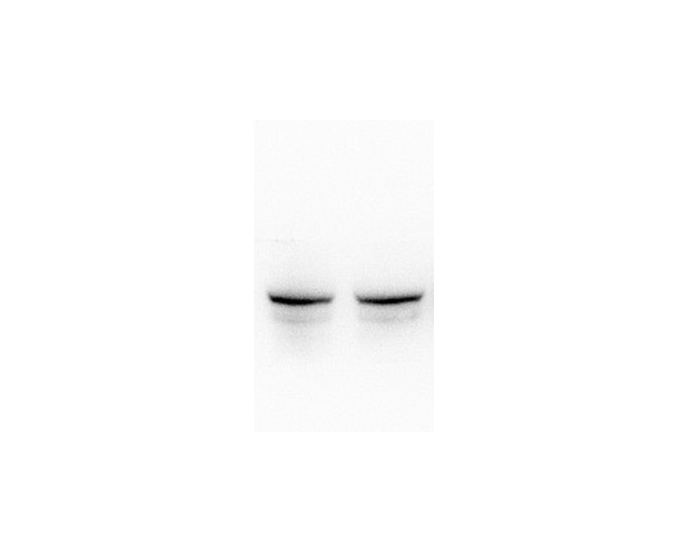

Supplement: Supplemental Information 1 [file peerj-10-13815-s001.zip › Raw data - Western blot bands/fig1/caspase3/caspase 3.tif]

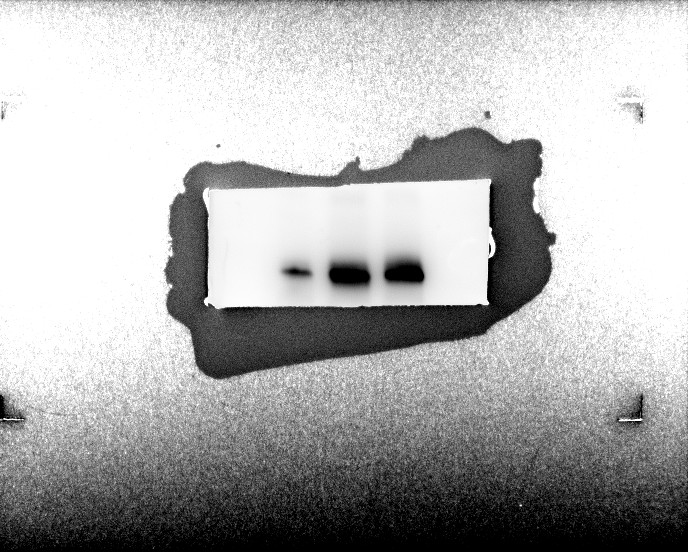

Supplement: Supplemental Information 1 [file peerj-10-13815-s001.zip › Raw data - Western blot bands/fig1/GAPDH/GAPDH1.jpg]

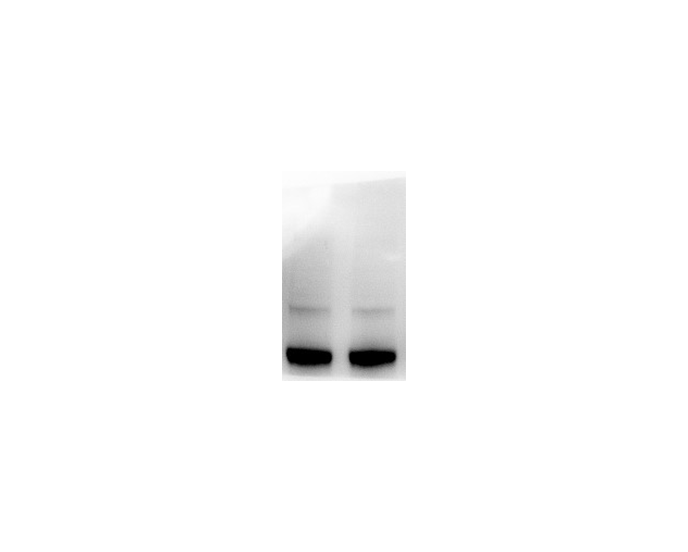

Supplement: Supplemental Information 1 [file peerj-10-13815-s001.zip › Raw data - Western blot bands/fig1/GAPDH/GAPDH2.tif]

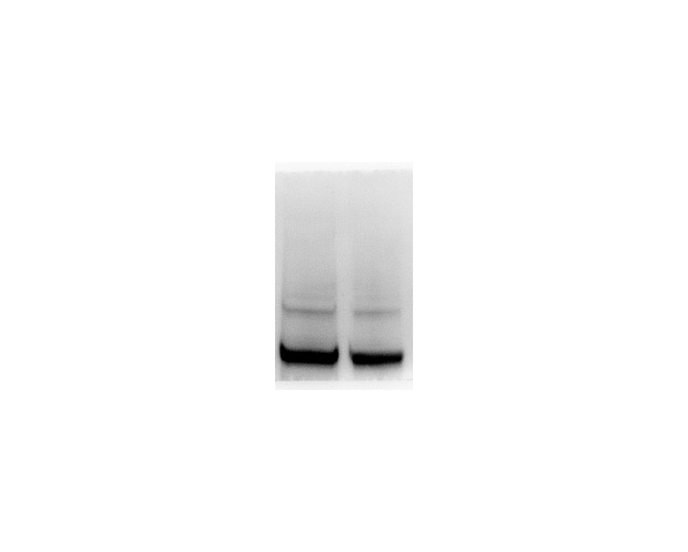

Supplement: Supplemental Information 1 [file peerj-10-13815-s001.zip › Raw data - Western blot bands/fig1/GAPDH/GAPDH3.tif]

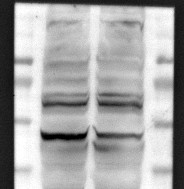

Supplement: Supplemental Information 1 [file peerj-10-13815-s001.zip › Raw data - Western blot bands/fig1/TRIM38/TRIM38 1.jpg]

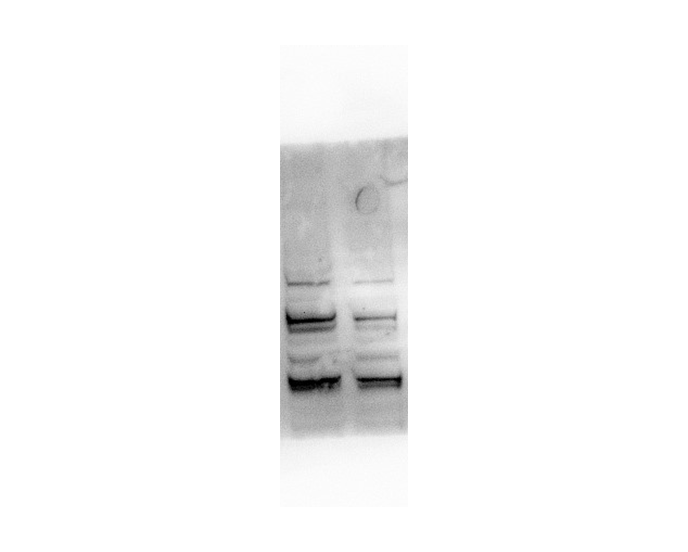

Supplement: Supplemental Information 1 [file peerj-10-13815-s001.zip › Raw data - Western blot bands/fig1/TRIM38/TRIM38 2.tif]

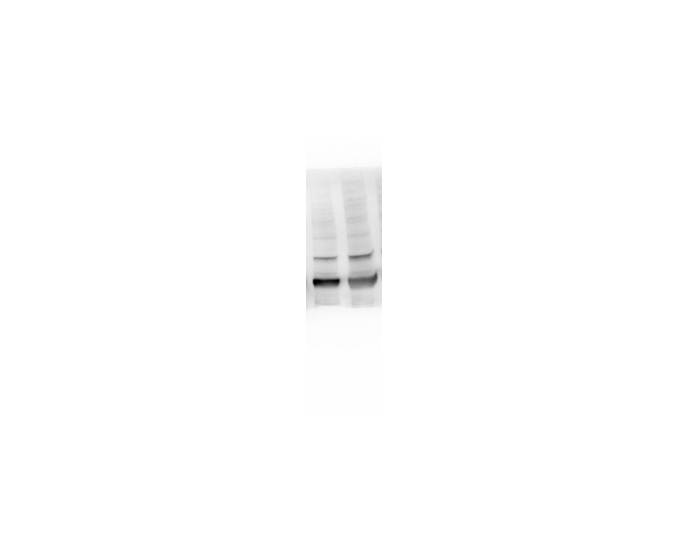

Supplement: Supplemental Information 1 [file peerj-10-13815-s001.zip › Raw data - Western blot bands/fig1/TRIM38/TRIM38 3.tif]

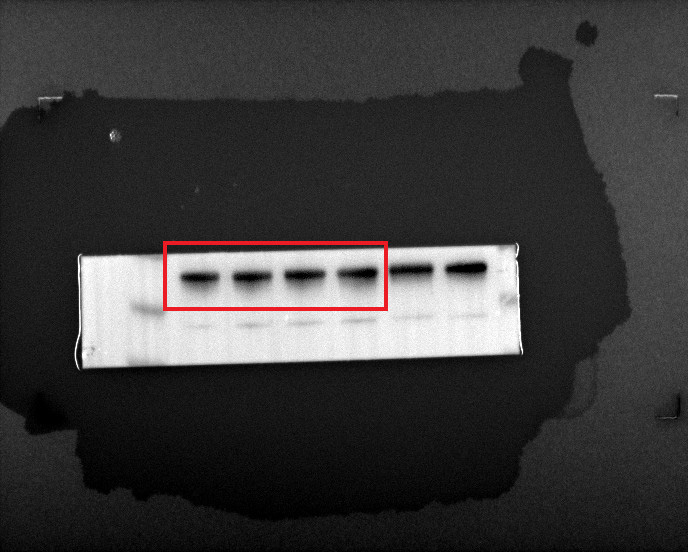

Supplement: Supplemental Information 1 [file peerj-10-13815-s001.zip › Raw data - Western blot bands/fig2/fig2A/GAPDH/GAPDH 1.jpg]

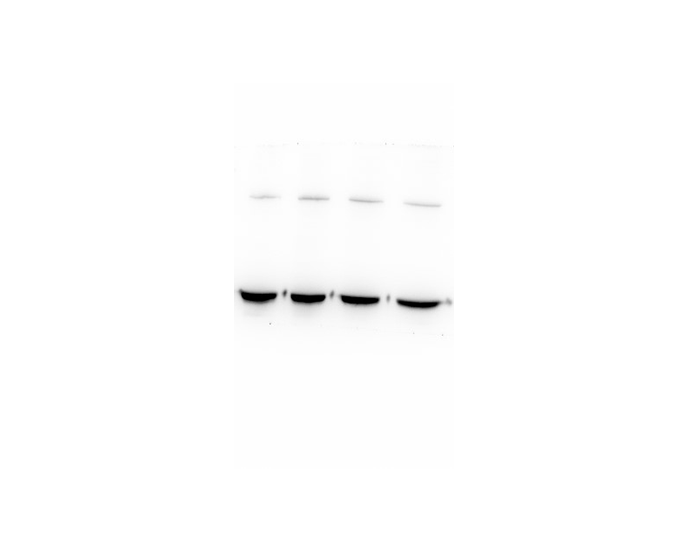

Supplement: Supplemental Information 1 [file peerj-10-13815-s001.zip › Raw data - Western blot bands/fig2/fig2A/GAPDH/GAPDH 2.tif]

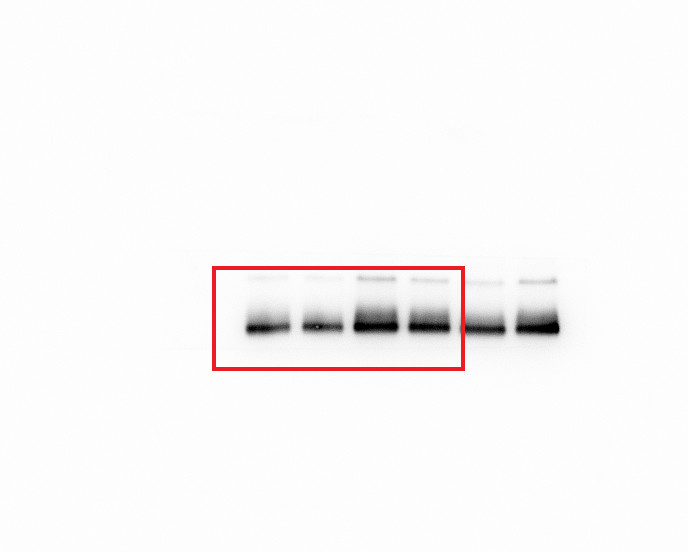

Supplement: Supplemental Information 1 [file peerj-10-13815-s001.zip › Raw data - Western blot bands/fig2/fig2A/TRIM38/TRIM38 1.jpg]

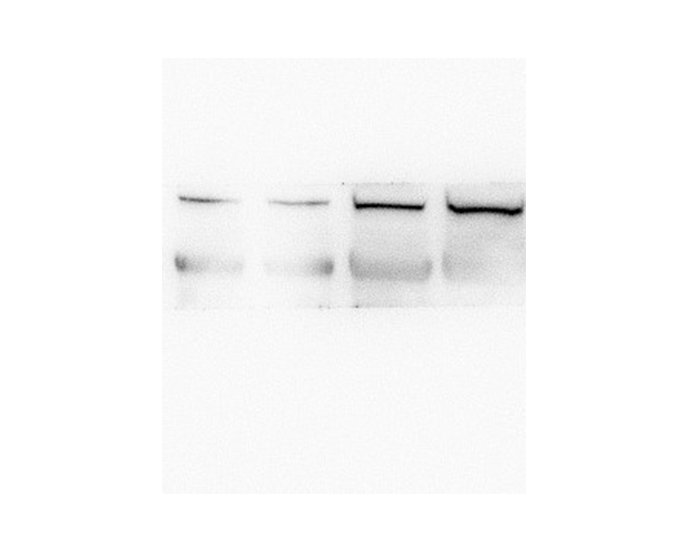

Supplement: Supplemental Information 1 [file peerj-10-13815-s001.zip › Raw data - Western blot bands/fig2/fig2A/TRIM38/TRIM38 2.tif]

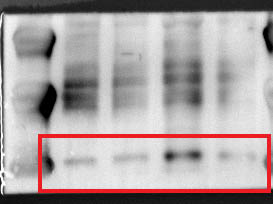

Supplement: Supplemental Information 1 [file peerj-10-13815-s001.zip › Raw data - Western blot bands/fig2/fig2B/bax/1.jpg]

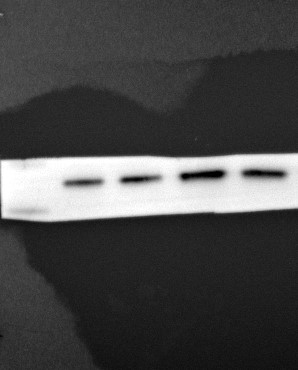

Supplement: Supplemental Information 1 [file peerj-10-13815-s001.zip › Raw data - Western blot bands/fig2/fig2B/bax/2.jpg]

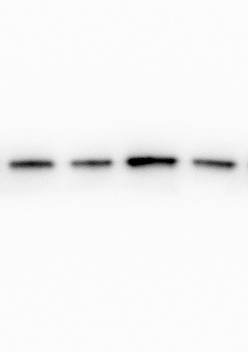

Supplement: Supplemental Information 1 [file peerj-10-13815-s001.zip › Raw data - Western blot bands/fig2/fig2B/bax/3.jpg]

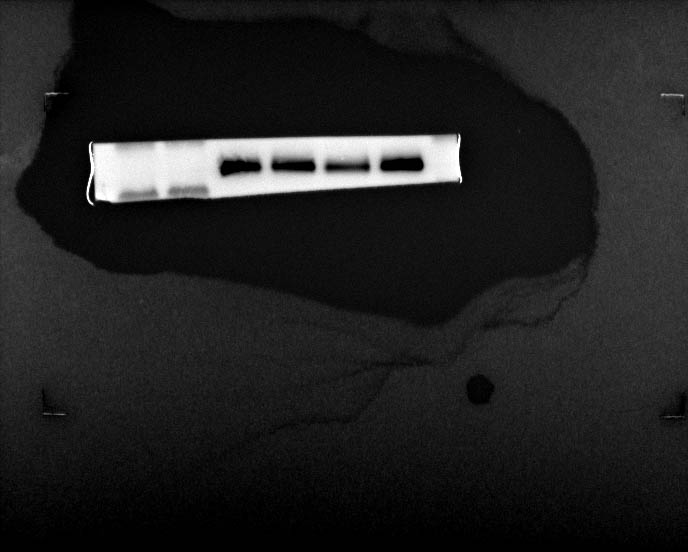

Supplement: Supplemental Information 1 [file peerj-10-13815-s001.zip › Raw data - Western blot bands/fig2/fig2B/bcl-2/1.jpg]

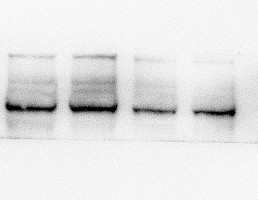

Supplement: Supplemental Information 1 [file peerj-10-13815-s001.zip › Raw data - Western blot bands/fig2/fig2B/bcl-2/2.jpg]

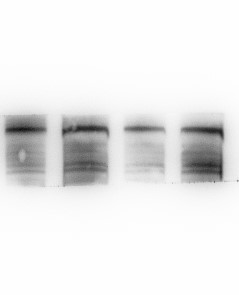

Supplement: Supplemental Information 1 [file peerj-10-13815-s001.zip › Raw data - Western blot bands/fig2/fig2B/bcl-2/3.jpg]

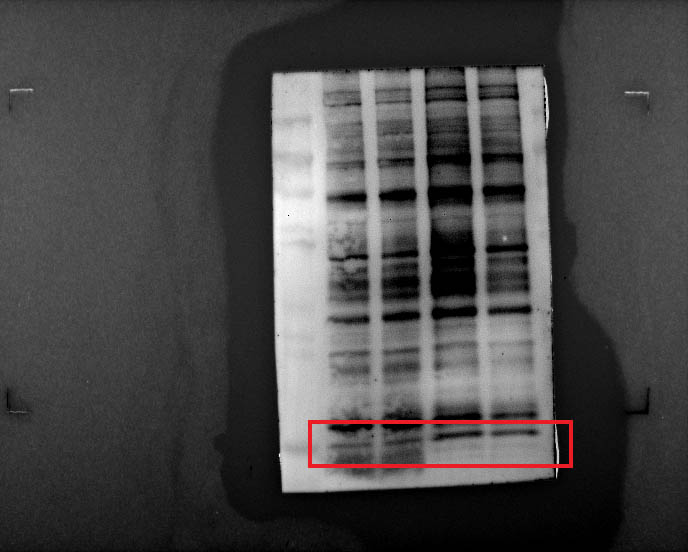

Supplement: Supplemental Information 1 [file peerj-10-13815-s001.zip › Raw data - Western blot bands/fig2/fig2B/c-caspase3/1.jpg]

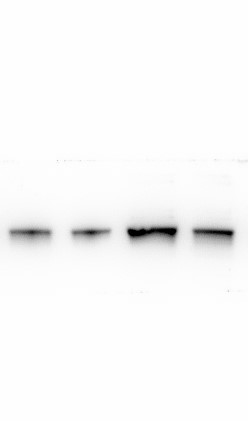

Supplement: Supplemental Information 1 [file peerj-10-13815-s001.zip › Raw data - Western blot bands/fig2/fig2B/c-caspase3/2.jpg]

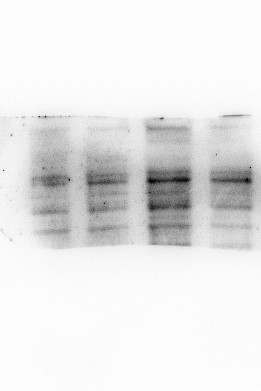

Supplement: Supplemental Information 1 [file peerj-10-13815-s001.zip › Raw data - Western blot bands/fig2/fig2B/c-caspase3/3.jpg]

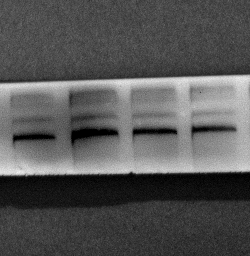

Supplement: Supplemental Information 1 [file peerj-10-13815-s001.zip › Raw data - Western blot bands/fig2/fig2B/caspase3/1.tif]

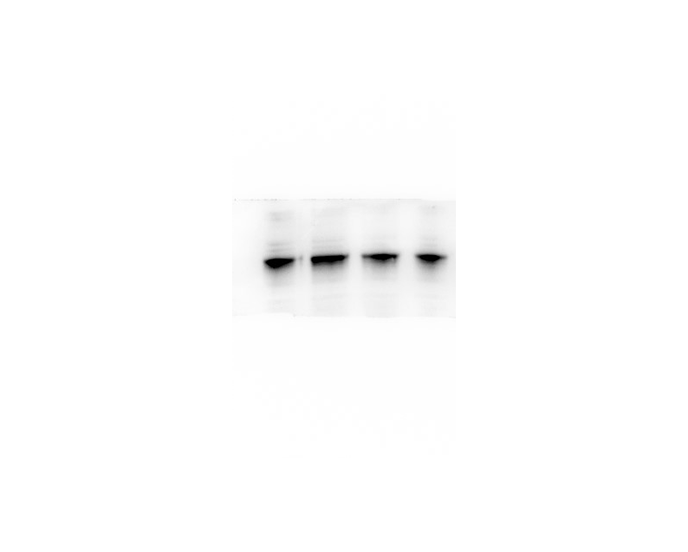

Supplement: Supplemental Information 1 [file peerj-10-13815-s001.zip › Raw data - Western blot bands/fig2/fig2B/caspase3/2.tif]

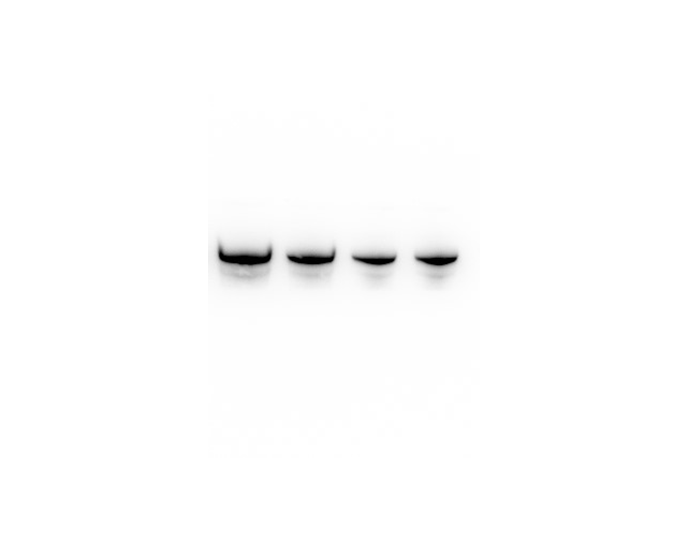

Supplement: Supplemental Information 1 [file peerj-10-13815-s001.zip › Raw data - Western blot bands/fig2/fig2B/caspase3/3.tif]

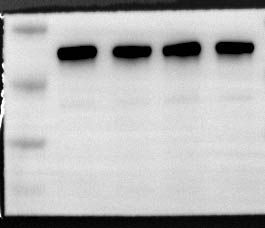

Supplement: Supplemental Information 1 [file peerj-10-13815-s001.zip › Raw data - Western blot bands/fig2/fig2B/GAPDH/1.jpg]

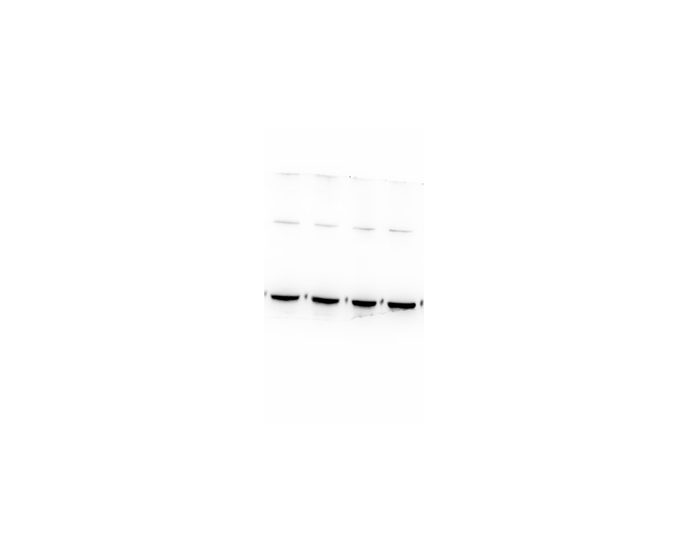

Supplement: Supplemental Information 1 [file peerj-10-13815-s001.zip › Raw data - Western blot bands/fig2/fig2B/GAPDH/2.tif]

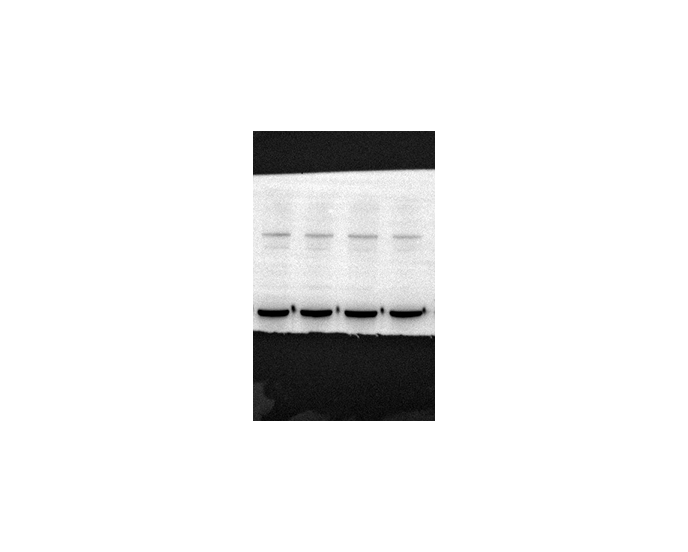

Supplement: Supplemental Information 1 [file peerj-10-13815-s001.zip › Raw data - Western blot bands/fig2/fig2B/GAPDH/3.tif]

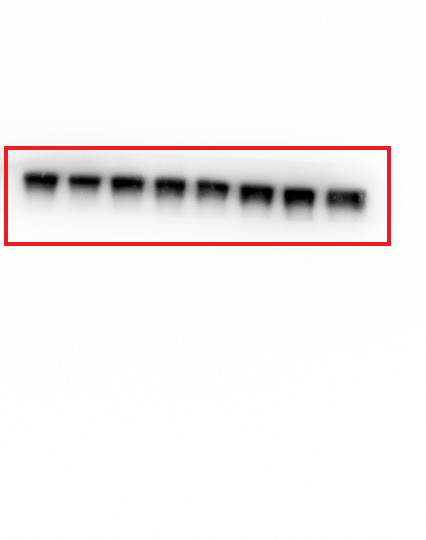

Supplement: Supplemental Information 1 [file peerj-10-13815-s001.zip › Raw data - Western blot bands/fig3/fig3A/GAPDH/GAPDH 1.jpg]

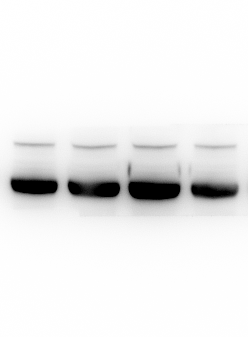

Supplement: Supplemental Information 1 [file peerj-10-13815-s001.zip › Raw data - Western blot bands/fig3/fig3A/GAPDH/GAPDH 2.tiff]

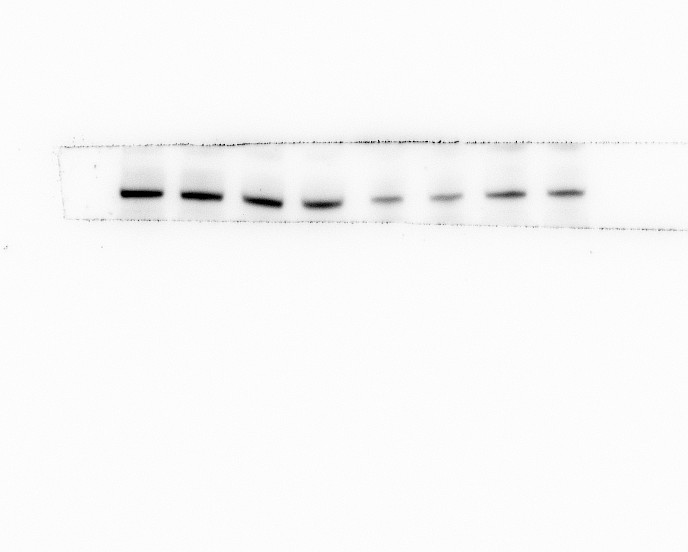

Supplement: Supplemental Information 1 [file peerj-10-13815-s001.zip › Raw data - Western blot bands/fig3/fig3A/TRIM38/TRIM38 1.jpg]

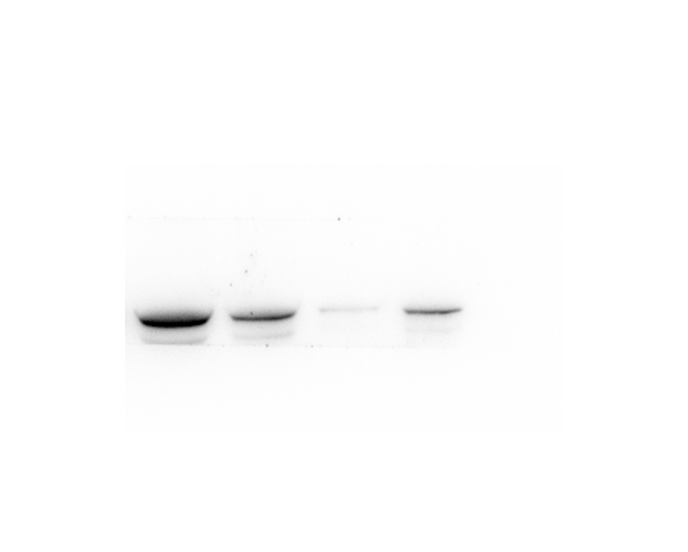

Supplement: Supplemental Information 1 [file peerj-10-13815-s001.zip › Raw data - Western blot bands/fig3/fig3A/TRIM38/TRIM38 2.tif]

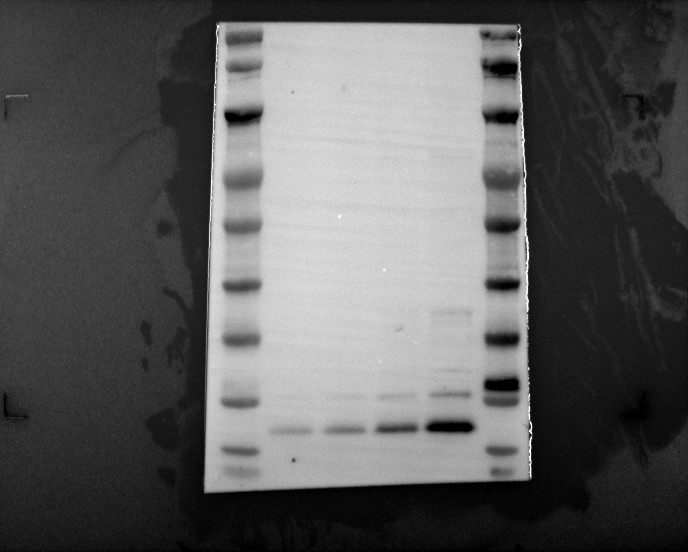

Supplement: Supplemental Information 1 [file peerj-10-13815-s001.zip › Raw data - Western blot bands/fig3/fig3B/bax/1.jpg]

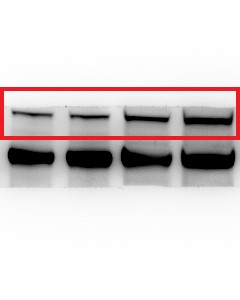

Supplement: Supplemental Information 1 [file peerj-10-13815-s001.zip › Raw data - Western blot bands/fig3/fig3B/bax/2.jpg]

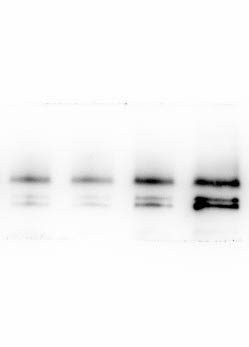

Supplement: Supplemental Information 1 [file peerj-10-13815-s001.zip › Raw data - Western blot bands/fig3/fig3B/bax/3.jpg]

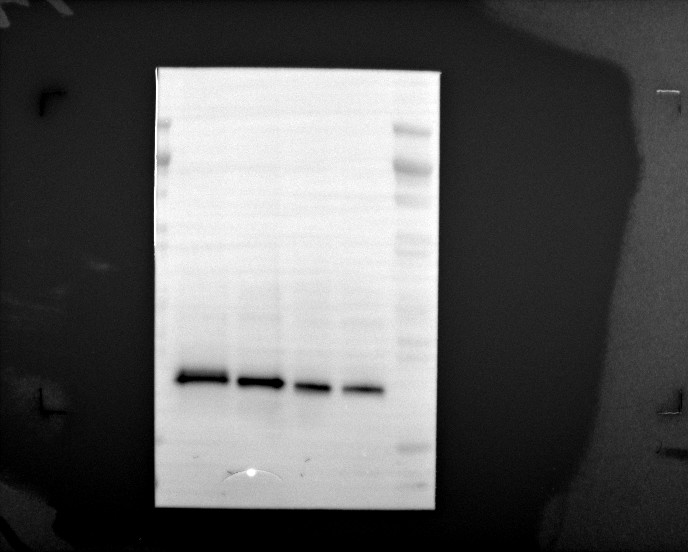

Supplement: Supplemental Information 1 [file peerj-10-13815-s001.zip › Raw data - Western blot bands/fig3/fig3B/bcl-2/1.jpg]

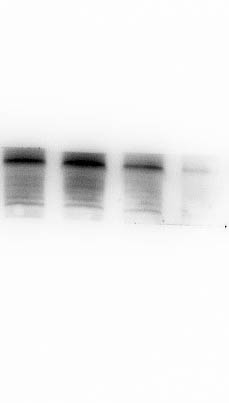

Supplement: Supplemental Information 1 [file peerj-10-13815-s001.zip › Raw data - Western blot bands/fig3/fig3B/bcl-2/2.jpg]

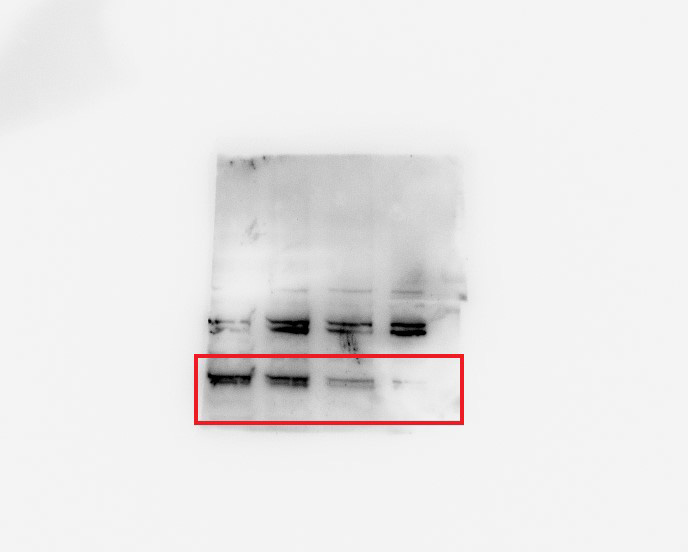

Supplement: Supplemental Information 1 [file peerj-10-13815-s001.zip › Raw data - Western blot bands/fig3/fig3B/bcl-2/3.jpg]

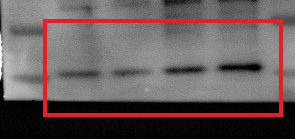

Supplement: Supplemental Information 1 [file peerj-10-13815-s001.zip › Raw data - Western blot bands/fig3/fig3B/c-caspase3/1.jpg]

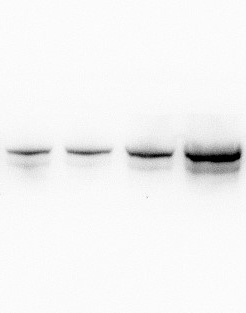

Supplement: Supplemental Information 1 [file peerj-10-13815-s001.zip › Raw data - Western blot bands/fig3/fig3B/c-caspase3/2.jpg]

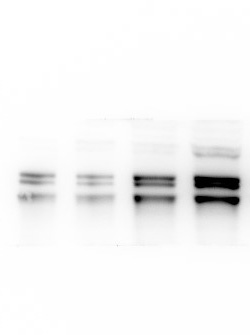

Supplement: Supplemental Information 1 [file peerj-10-13815-s001.zip › Raw data - Western blot bands/fig3/fig3B/c-caspase3/3.jpg]

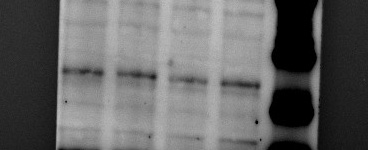

Supplement: Supplemental Information 1 [file peerj-10-13815-s001.zip › Raw data - Western blot bands/fig3/fig3B/caspase3/1.jpg]

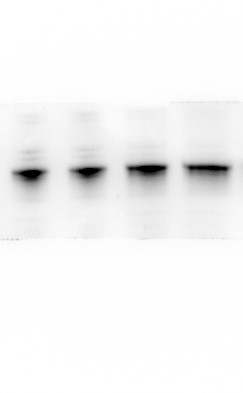

Supplement: Supplemental Information 1 [file peerj-10-13815-s001.zip › Raw data - Western blot bands/fig3/fig3B/caspase3/2.jpg]

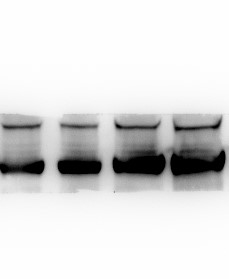

Supplement: Supplemental Information 1 [file peerj-10-13815-s001.zip › Raw data - Western blot bands/fig3/fig3B/caspase3/3.jpg]

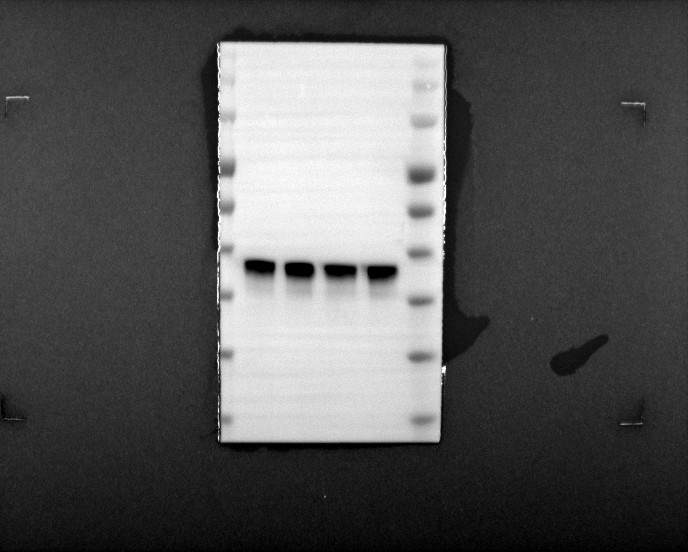

Supplement: Supplemental Information 1 [file peerj-10-13815-s001.zip › Raw data - Western blot bands/fig3/fig3B/GAPDH/GAPDH 1.jpg]

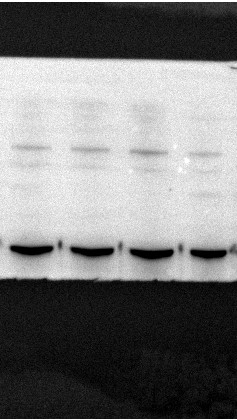

Supplement: Supplemental Information 1 [file peerj-10-13815-s001.zip › Raw data - Western blot bands/fig3/fig3B/GAPDH/GAPDH 2.jpg]

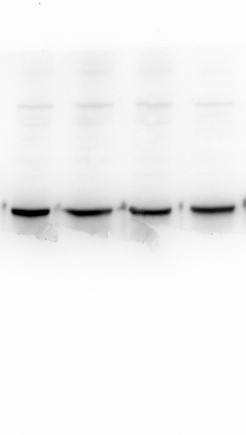

Supplement: Supplemental Information 1 [file peerj-10-13815-s001.zip › Raw data - Western blot bands/fig3/fig3B/GAPDH/GAPDH 3.jpg]

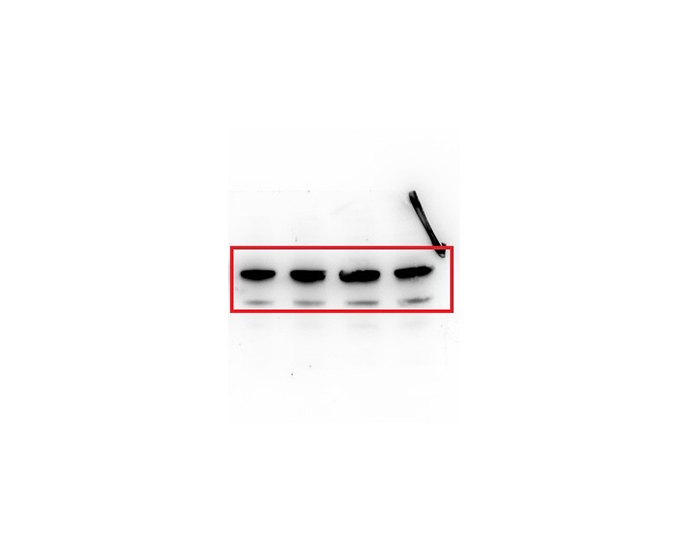

Supplement: Supplemental Information 1 [file peerj-10-13815-s001.zip › Raw data - Western blot bands/fig4/fig4A/GAPDH/1.tif]

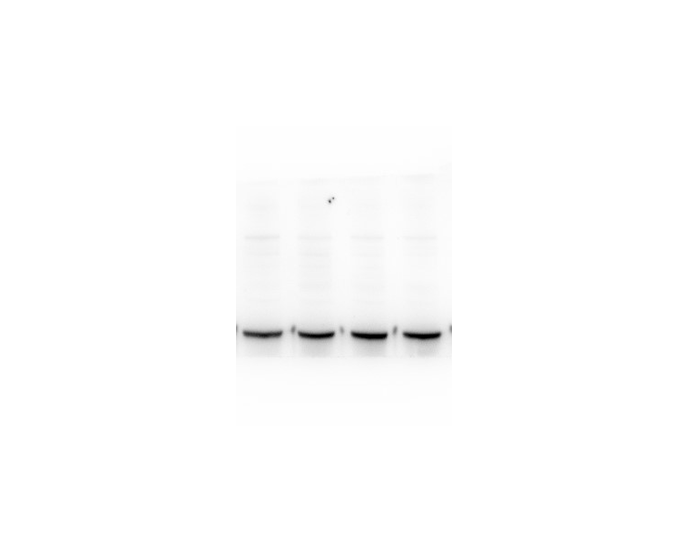

Supplement: Supplemental Information 1 [file peerj-10-13815-s001.zip › Raw data - Western blot bands/fig4/fig4A/GAPDH/2.tif]

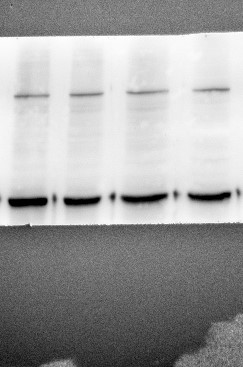

Supplement: Supplemental Information 1 [file peerj-10-13815-s001.zip › Raw data - Western blot bands/fig4/fig4A/GAPDH/3.jpg]

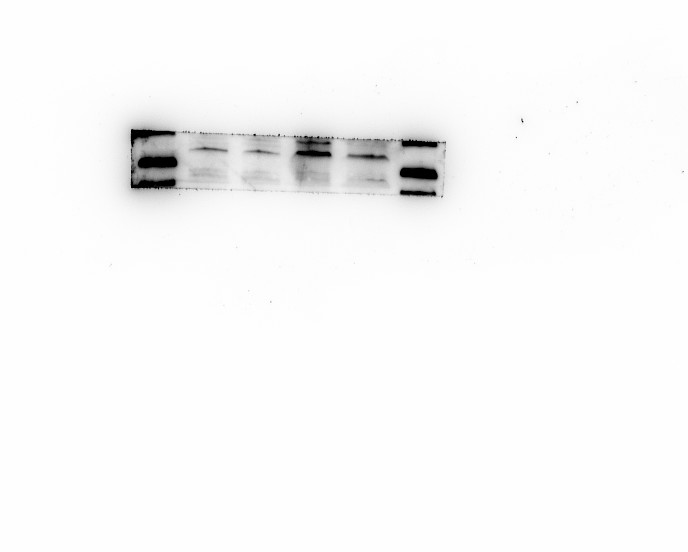

Supplement: Supplemental Information 1 [file peerj-10-13815-s001.zip › Raw data - Western blot bands/fig4/fig4A/IL-1β/1.jpg]

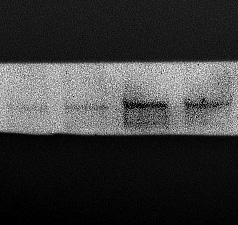

Supplement: Supplemental Information 1 [file peerj-10-13815-s001.zip › Raw data - Western blot bands/fig4/fig4A/IL-1β/2.jpg]

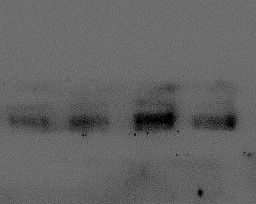

Supplement: Supplemental Information 1 [file peerj-10-13815-s001.zip › Raw data - Western blot bands/fig4/fig4A/IL-1β/3.jpg]

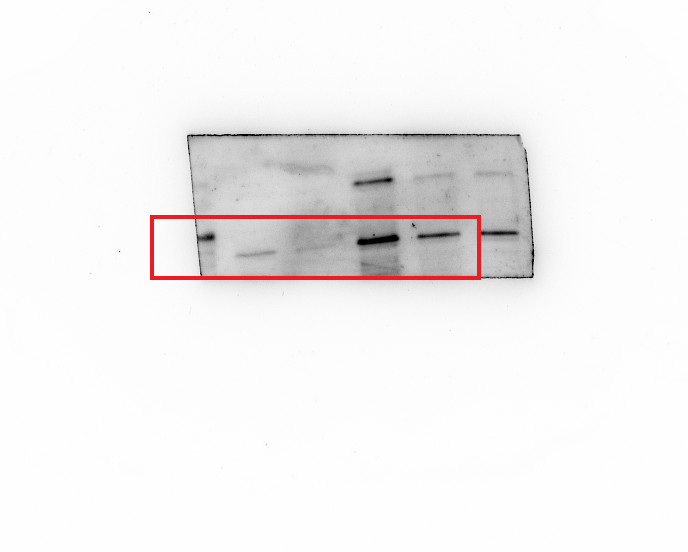

Supplement: Supplemental Information 1 [file peerj-10-13815-s001.zip › Raw data - Western blot bands/fig4/fig4A/IL-6/1.jpg]

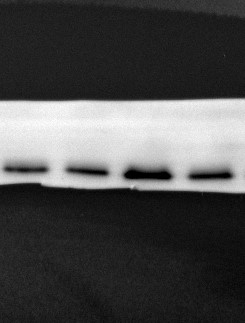

Supplement: Supplemental Information 1 [file peerj-10-13815-s001.zip › Raw data - Western blot bands/fig4/fig4A/IL-6/2.jpg]

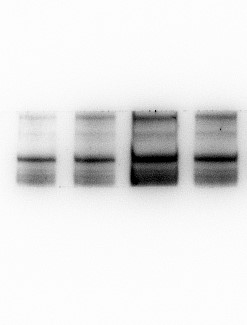

Supplement: Supplemental Information 1 [file peerj-10-13815-s001.zip › Raw data - Western blot bands/fig4/fig4A/IL-6/3.jpg]

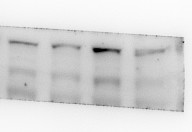

Supplement: Supplemental Information 1 [file peerj-10-13815-s001.zip › Raw data - Western blot bands/fig4/fig4A/TNF-α/TNF-α.jpg]

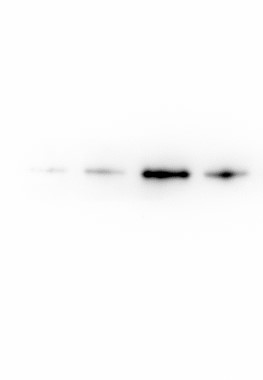

Supplement: Supplemental Information 1 [file peerj-10-13815-s001.zip › Raw data - Western blot bands/fig4/fig4A/TNF-α/TNF-α2.jpg]

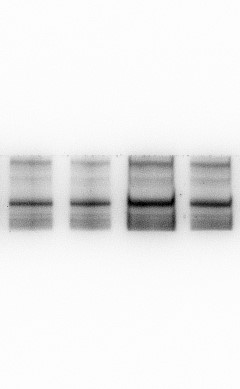

Supplement: Supplemental Information 1 [file peerj-10-13815-s001.zip › Raw data - Western blot bands/fig4/fig4A/TNF-α/TNF-α3.jpg]

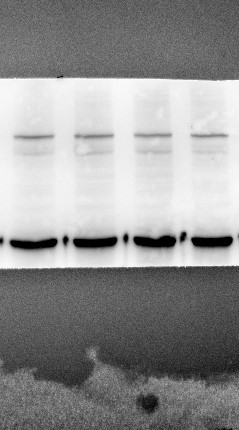

Supplement: Supplemental Information 1 [file peerj-10-13815-s001.zip › Raw data - Western blot bands/fig4/fig4B/GAPDH/GAPDH 2.jpg]

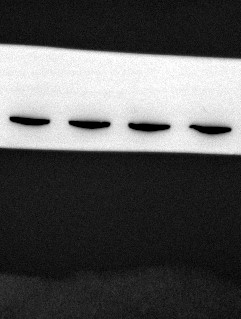

Supplement: Supplemental Information 1 [file peerj-10-13815-s001.zip › Raw data - Western blot bands/fig4/fig4B/GAPDH/GAPDH 3.jpg]

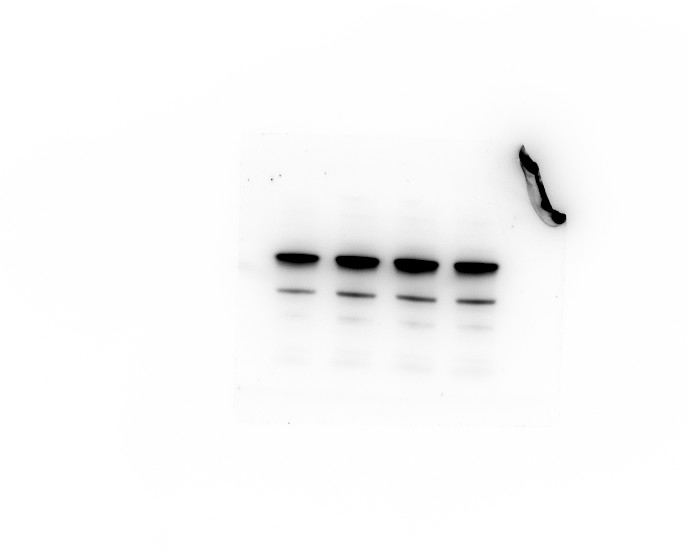

Supplement: Supplemental Information 1 [file peerj-10-13815-s001.zip › Raw data - Western blot bands/fig4/fig4B/GAPDH/GAPDH.jpg]

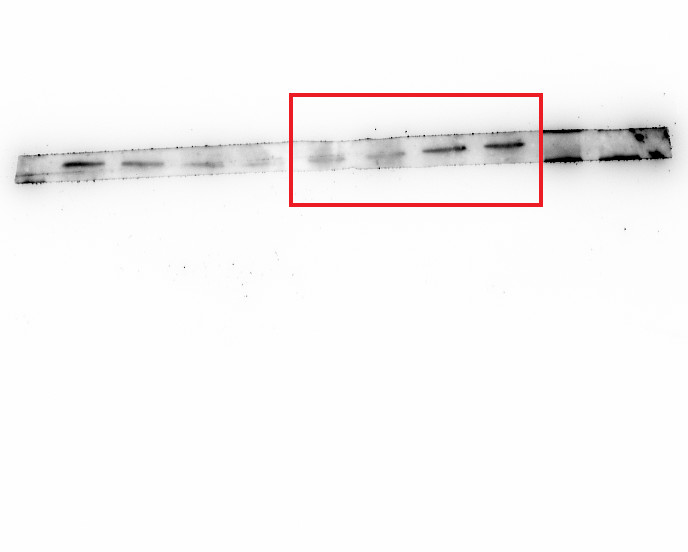

Supplement: Supplemental Information 1 [file peerj-10-13815-s001.zip › Raw data - Western blot bands/fig4/fig4B/IL-1β/IL-1β.jpg]

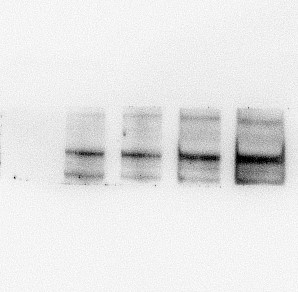

Supplement: Supplemental Information 1 [file peerj-10-13815-s001.zip › Raw data - Western blot bands/fig4/fig4B/IL-1β/IL-1β2.jpg]

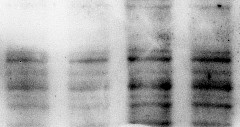

Supplement: Supplemental Information 1 [file peerj-10-13815-s001.zip › Raw data - Western blot bands/fig4/fig4B/IL-1β/IL-1β3.jpg]

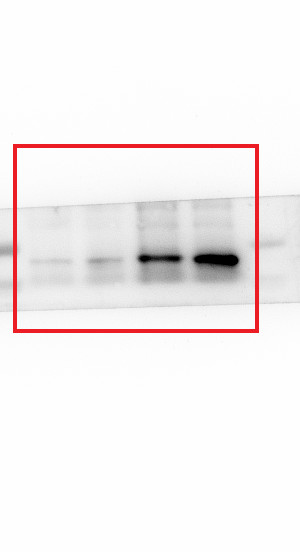

Supplement: Supplemental Information 1 [file peerj-10-13815-s001.zip › Raw data - Western blot bands/fig4/fig4B/IL-6/1.jpg]

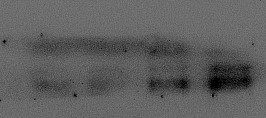

Supplement: Supplemental Information 1 [file peerj-10-13815-s001.zip › Raw data - Western blot bands/fig4/fig4B/IL-6/2.jpg]

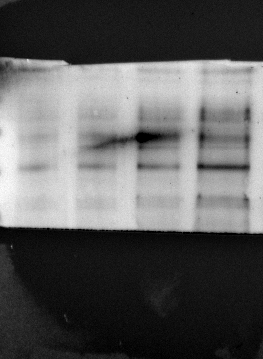

Supplement: Supplemental Information 1 [file peerj-10-13815-s001.zip › Raw data - Western blot bands/fig4/fig4B/IL-6/3.tiff]

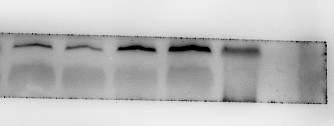

Supplement: Supplemental Information 1 [file peerj-10-13815-s001.zip › Raw data - Western blot bands/fig4/fig4B/TNF-α/TNF-α.jpg]

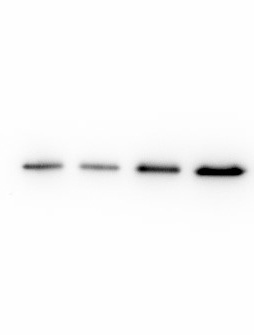

Supplement: Supplemental Information 1 [file peerj-10-13815-s001.zip › Raw data - Western blot bands/fig4/fig4B/TNF-α/TNF-α1.jpg]

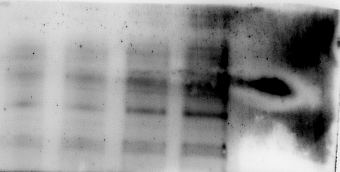

Supplement: Supplemental Information 1 [file peerj-10-13815-s001.zip › Raw data - Western blot bands/fig4/fig4B/TNF-α/TNF-α2.tiff]

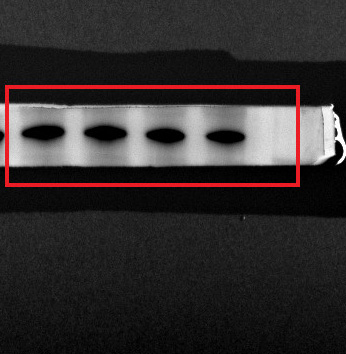

Supplement: Supplemental Information 1 [file peerj-10-13815-s001.zip › Raw data - Western blot bands/fig4/fig4E/GAPDH/GAPDH.jpg]

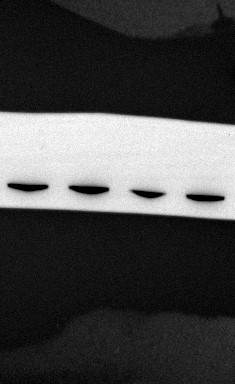

Supplement: Supplemental Information 1 [file peerj-10-13815-s001.zip › Raw data - Western blot bands/fig4/fig4E/GAPDH/GAPDH2.jpg]

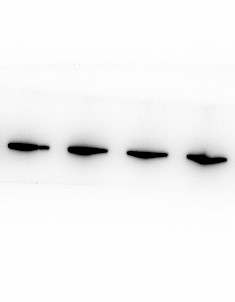

Supplement: Supplemental Information 1 [file peerj-10-13815-s001.zip › Raw data - Western blot bands/fig4/fig4E/GAPDH/GAPDH3.jpg]

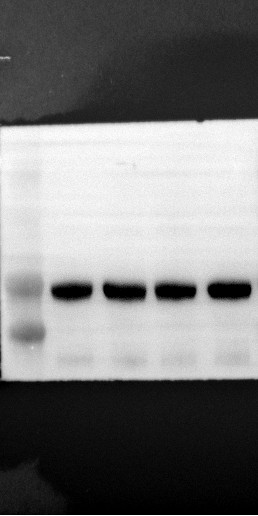

Supplement: Supplemental Information 1 [file peerj-10-13815-s001.zip › Raw data - Western blot bands/fig4/fig4E/IKK/1.jpg]

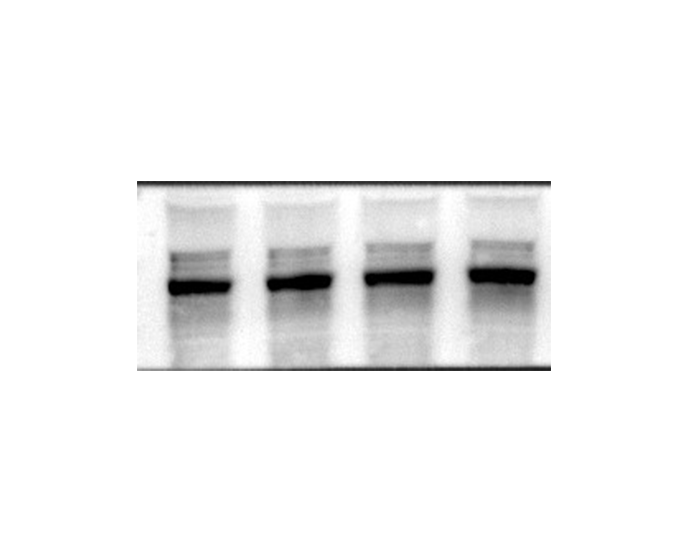

Supplement: Supplemental Information 1 [file peerj-10-13815-s001.zip › Raw data - Western blot bands/fig4/fig4E/IKK/2.tif]

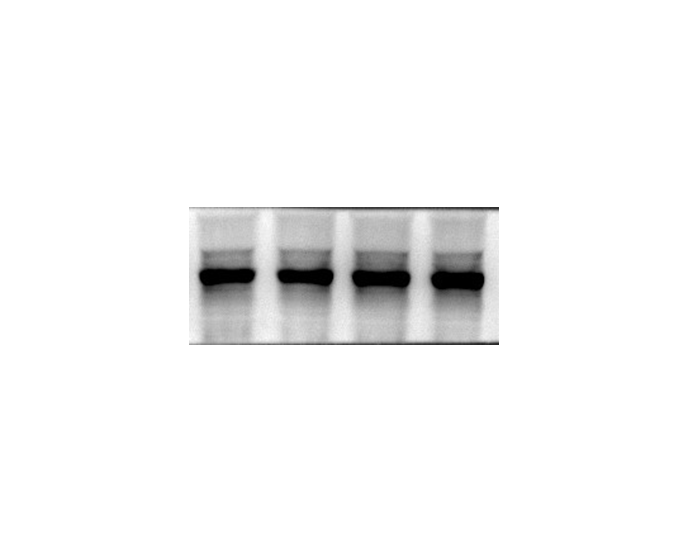

Supplement: Supplemental Information 1 [file peerj-10-13815-s001.zip › Raw data - Western blot bands/fig4/fig4E/IKK/3.tif]

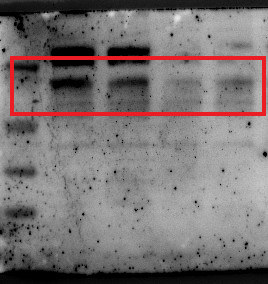

Supplement: Supplemental Information 1 [file peerj-10-13815-s001.zip › Raw data - Western blot bands/fig4/fig4E/IκBα/IκBα.jpg]

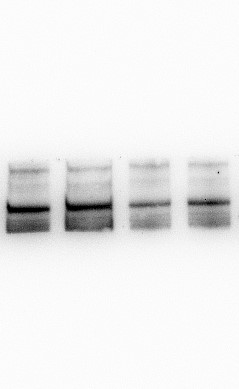

Supplement: Supplemental Information 1 [file peerj-10-13815-s001.zip › Raw data - Western blot bands/fig4/fig4E/IκBα/IκBα2.jpg]

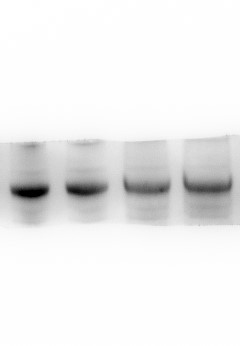

Supplement: Supplemental Information 1 [file peerj-10-13815-s001.zip › Raw data - Western blot bands/fig4/fig4E/IκBα/IκBα3.jpg]

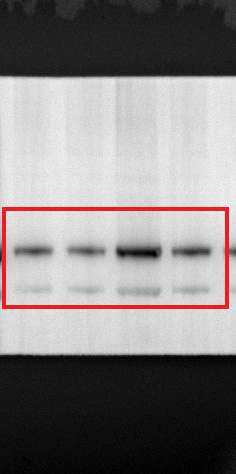

Supplement: Supplemental Information 1 [file peerj-10-13815-s001.zip › Raw data - Western blot bands/fig4/fig4E/p-IKK/1.jpg]

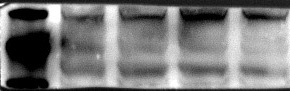

Supplement: Supplemental Information 1 [file peerj-10-13815-s001.zip › Raw data - Western blot bands/fig4/fig4E/p-IKK/2.jpg]

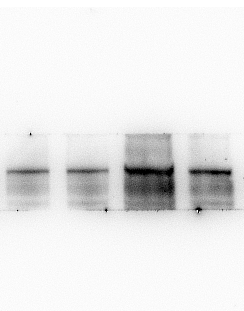

Supplement: Supplemental Information 1 [file peerj-10-13815-s001.zip › Raw data - Western blot bands/fig4/fig4E/p-IKK/3.tiff]

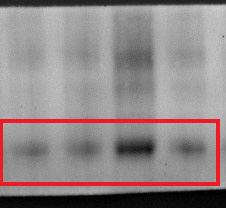

Supplement: Supplemental Information 1 [file peerj-10-13815-s001.zip › Raw data - Western blot bands/fig4/fig4E/p-IκBα/p-IκBα.jpg]

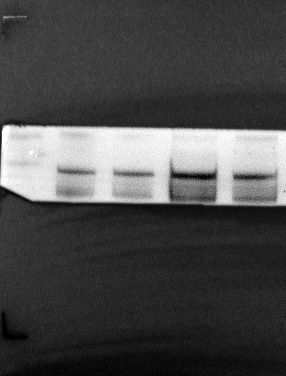

Supplement: Supplemental Information 1 [file peerj-10-13815-s001.zip › Raw data - Western blot bands/fig4/fig4E/p-IκBα/p-IκBα2.jpg]

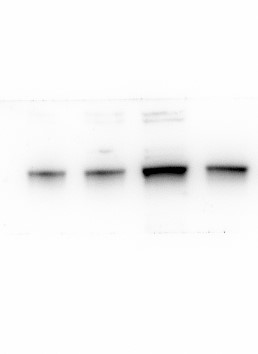

Supplement: Supplemental Information 1 [file peerj-10-13815-s001.zip › Raw data - Western blot bands/fig4/fig4E/p-IκBα/p-IκBα3.jpg]

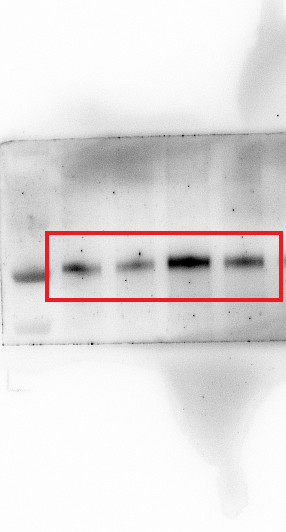

Supplement: Supplemental Information 1 [file peerj-10-13815-s001.zip › Raw data - Western blot bands/fig4/fig4E/p-p65/1.jpg]

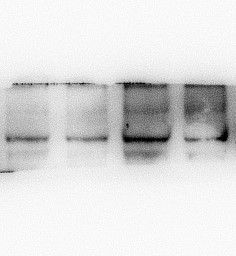

Supplement: Supplemental Information 1 [file peerj-10-13815-s001.zip › Raw data - Western blot bands/fig4/fig4E/p-p65/2.jpg]

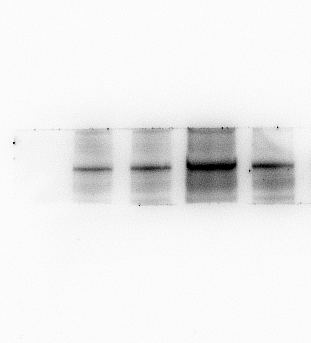

Supplement: Supplemental Information 1 [file peerj-10-13815-s001.zip › Raw data - Western blot bands/fig4/fig4E/p-p65/3.tiff]

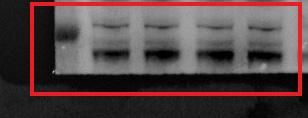

Supplement: Supplemental Information 1 [file peerj-10-13815-s001.zip › Raw data - Western blot bands/fig4/fig4E/p65/1.jpg]

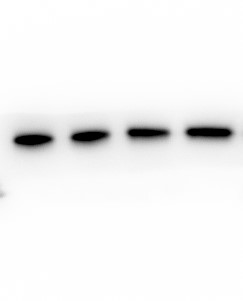

Supplement: Supplemental Information 1 [file peerj-10-13815-s001.zip › Raw data - Western blot bands/fig4/fig4E/p65/2.jpg]
